# Neutrophil-macrophage crosstalk via NETs–IL-17/VEGF/S100A9 axis promotes hepatocellular carcinoma progression

**DOI:** 10.1186/s13046-025-03618-x

**Published:** 2025-12-30

**Authors:** Rong Wu, Rui Wu, Xuehua Kong, Xuanyi Wang, Yaqian Duan, Shiyu Cao, Shan Yu, Yuqing Zhao, Shue Li, Jingying Zhou, Liang Duan

**Affiliations:** 1 Department of Laboratory Medicine, The Second Affiliated Hospital of Chongqing Medical University, No.74 linjiang Road, Yu Zhong District, Chongqing, 400010 China; 2 Department of Laboratory Medicine, The First Affiliated Hospital of Chongqing Medical University, Chongqing, 400000 China; 3 Department of Pathology, The Second Affiliated Hospital of Chongqing Medical University, Chongqing, 400010 China; 4 Department of Critical Care Medicine, The Second Affiliated Hospital of Chongqing Medical University, Chongqing, 400010 China; 5 School of Biomedical Sciences, The Chinese University of Hong Kong, Lo Kwee-Seong Integrated Biomedical Sciences Building, Area 39, Shatin, Hong Kong 999077 China

**Keywords:** NETs, VEGF, HCC, Neutrophil, Macrophage

## Abstract

**Background:**

Tumor-associated neutrophils and macrophages are key components of the hepatocellular carcinoma (HCC) microenvironment. However, the interplay between them and its contribution to HCC progression remain unclear.

**Methods:**

Bioinformatic analysis of TCGA datasets and clinical HCC samples was used to evaluate neutrophil extracellular trap (NETs) levels and macrophage polarization. Co-culture of neutrophils, macrophages, and HCC cells, along with molecular analysis and in vivo mouse models, were employed to dissect the mechanisms underlying NETs-mediated macrophage reprogramming and tumor progression.

**Results:**

NETs were significantly elevated in HCC patients, particularly in advanced and metastatic stages, which were positively correlated with intrahepatic M2 macrophage infiltration and M2d subset-associated cytokines in blood. In vitro, NETs promoted M2d polarization in the presence of HCC cells via IL-17R/NF-κB signaling activated by IL-17 carried within NETs, which subsequently enhanced angiogenesis, migration, invasion, and epithelial-mesenchymal transition; these effects were partially reversed by IL-17R inhibition. In vivo, NETs-induced M2d polarization accelerated tumor growth, angiogenesis, and metastasis, whereas IL-17R blockade attenuated these pro-tumor effects. Moreover, M2d macrophages indirectly promoted NETs formation by upregulating HCC cell-derived S100A9 through VEGF-NF-κB signaling, establishing a positive feedback loop between neutrophils and macrophages. Furthermore, IL-17 carried by NETs (NETs-IL-17) demonstrated strong predictive value for extrahepatic metastasis in HCC, with an area under the ROC curve (AUC) of 0.89.

**Conclusions:**

A positive feedback loop between neutrophils and macrophages via the NETs-IL-17/VEGF/S100A9 axis accelerates HCC progression and metastasis. More importantly, NETs-IL-17 exhibited potential as an alternative biomarker for predicting extrahepatic metastasis in HCC.

**Supplementary Information:**

The online version contains supplementary material available at 10.1186/s13046-025-03618-x.

## Introduction

Hepatocellular carcinoma (HCC), the most common primary liver malignancy, predominantly develops in the context of chronic liver inflammation caused by hepatitis B or C virus infection, alcohol abuse, or non-alcoholic steatohepatitis [1]. Persistent hepatic inflammation drives progressive fibrosis and cirrhosis, ultimately predisposing hepatocytes to malignant transformation [2]. The HCC microenvironment is characterized by a complex stromal architecture enriched with immune and inflammatory cells. Among these, neutrophils and macrophages have recently emerged as pivotal regulators of tumor-promoting inflammation and immune evasion [3]. Nevertheless, the molecular mechanisms orchestrating neutrophil–macrophage crosstalk within the HCC microenvironment and their contribution to disease progression remain poorly understood.

Neutrophil extracellular traps (NETs) are web-like structures composed of DNA, histones, and antimicrobial proteins released by activated neutrophils in response to infection or inflammation [4]. Although initially recognized for trapping and neutralizing pathogens, emerging evidence indicates that NETs also contribute to cancer progression by enhancing metastasis, modulating immune responses, and promoting angiogenesis [5]. Our previous studies demonstrated that NETs, released by neutrophils in the HCC microenvironment, can directly promote HCC cell growth and metastasis [6]. Recently, NETs carry multiple damage-associated molecular patterns (DAMPs) that can differentially modulate both tumor and immune cells. For instance, NETs-associated HMGB1 can activate monocytes and macrophages [7]; NETs-associated DNA acts as a chemotactic signal by binding to cancer cell-expressed CCDC25, thereby promoting cancer cell motility [8]; and NETs-associated histones can directly promote Th17 cell differentiation via TLR2 signaling [9]. Collectively, these findings highlight that NETs serve as carriers of DAMPs that regulate the function of both cancer cells and immune cells within the tumor microenvironment (TME), providing a mechanistic basis for their role in HCC progression.

Macrophages within the TME exhibit remarkable plasticity and can polarize into two main phenotypes: pro-inflammatory M1 macrophages and pro-tumor M2 macrophages [10]. In response to particular triggers and changes in gene expression within the TME, M2 macrophages can be subdivided into the functional subsets M2a, M2b, M2c, and M2d, in which M2d has gained interest for its contribution to tumor progression, angiogenesis, and immunosuppression, characterized by elevated secretion of IL-10, TGF-β, and VEGF [11, 12]. M2 macrophages are frequently enriched in the TME of various cancers, including HCC, where they establish a supportive niche for tumor cells [13, 14]. Notably, DAMPs are known to influence macrophage polarization and may contribute to sustaining the M2 phenotype, thereby facilitating tumor progression [15]. Given that NETs carry multiple DAMPs capable of modulating immune cells, it remains unclear whether NETs in the HCC microenvironment drive macrophage polarization through specific DAMPs and promote cancer progression. Moreover, once polarized into the M2 phenotype, macrophages secrete various cytokines and signaling molecules that can remodel the immune landscape and further support tumor growth [16]. Whether these macrophage-derived factors, in turn, influence neutrophil NET formation, establishing a reciprocal crosstalk between neutrophils and macrophages that drives HCC progression, is yet to be elucidated.

In this study, we explored the molecular mechanisms underlying the interaction between neutrophils and macrophages in the HCC microenvironment and their impact on tumor progression. Our study demonstrates that neutrophil-derived NETs carry IL-17 in HCC, which binds to IL-17R on macrophages, inducing their polarization into the M2d phenotype. This M2d polarization promotes the release of VEGF, which in turn triggers the secretion of S100A9 from HCC cells. S100A9 then stimulates further NET formation, establishing a positive feedback loop between neutrophils and macrophages via the NETs-IL-17/VEGF/S100A9 axis that ultimately facilitates HCC growth and metastasis. Our findings reveal a novel mechanism of immune crosstalk in the HCC microenvironment and suggest that disrupting this axis may offer a promising therapeutic strategy.

## Materials and methods

### Patients and clinical specimens

Peripheral blood samples were collected from 32 healthy controls (HCs) and 83 clinically diagnosed HCC patients at the Second Affiliated Hospital of Chongqing Medical University (Chongqing, China) between December 2024 and July 2025. In addition, six normal liver biopsy specimens were collected from HCs who underwent a biopsy to exclude malignancy. Tumor tissues were collected from eight HCC patients who underwent surgical resection. None of the HCC patients received chemotherapy or radiotherapy prior to surgery, and written informed consent was obtained from all participants. The study was approved by the Institutional Ethics Committee of The Second Affiliated Hospital of Chongqing Medical University in accordance with the Declaration of Helsinki (No. 2024 − 86). The characteristics of the enrolled individuals are summarized in Table 1.

**Table 1:** The clinical characteristics of enrolled individuals in this study

Characteristic	Peripheral blood specimen		Tissue specimen	
	HCC (*n* = 83)	HCs (*n* = 32)	HCC (*n* = 8)	Normal (*n* = 6)
Age, years, n (%)				
< 60	52 (62.65)	18 (56.25)	5 (62.50)	4 (66.67)
≥ 60	31 (37.35)	14 (43.75)	3 (37.50)	2 (33.33)
Gender, n (%)				
Male	54 (65.06)	19 (59.38)	5 (62.50)	4 (66.67)
Female	29 (34.94)	13 (40.62)	3 (37.50)	2 (33.33)
TNM stage, n (%)				
Ⅰ	16 (19.28)	N/A	1 (12.50)	N/A
Ⅱ	24 (28.92)	N/A	3 (37.50)	N/A
Ⅲ	21 (25.30)	N/A	2 (25.00)	N/A
Ⅳ	22 (26.50)	N/A	2 (25.00)	N/A
Tumor number, n (%)				
Solitary	57 (68.67)	N/A	6 (75.00)	N/A
Multiple	26 (31.33)	N/A	2 (25.00)	N/A
Tumor diameter, mm, n (%)				
< 50	67 (80.72)	N/A	5 (62.50)	N/A
≥ 50	16 (19.28)	N/A	3 (37.50)	N/A
Metastasis, n (%)				
Absent	44 (53.01)	N/A	5 (62.50)	N/A
Present	39 (46.99)	N/A	3 (37.50)	N/A
Extrahepatic metastasis status in total metastatic cases, n (%)				
Absent	21 (53.84)	N/A	1 (33.30)	N/A
Present	18 (46.16)	N/A	2 (66.70)	N/A

*Abbreviations*: *N/A* Not applicable, *n* Number of samples, *HCC* Hepatocellular Carcinoma

### Cells

The human liver normal cell line THLE-2, human HCC cell lines HuH-7 and HepG2, mouse HCC cell line Hepa1-6, human monocytic cell line THP-1, mouse macrophage cell line RAW264.7, and human umbilical vein endothelial cell line HUVEC were obtained from the central laboratory of the Second Affiliated Hospital of Chongqing Medical University (Chongqing, China). All cell lines were cultured in Dulbecco’s modified Eagle’s medium (DMEM, Gibco, USA) or RPMI 1640 medium (Gibco, USA) supplemented with 10% fetal bovine serum (FBS, Cellmax, China) and 1% penicillin-streptomycin (Beyotime, Shanghai, China) at 37 °C in a humidified atmosphere containing 5% CO₂.

THP-1 monocytes were cultured in complete RPMI-1640 medium and differentiated into M0 macrophages by treatment with phorbol 12-myristate 13-acetate (PMA, IP1010, Solarbio, China) at 100 ng/mL for 24 h. After PMA removal, cells were cultured in fresh complete medium for an additional 24 h to obtain THP-1-derived M0 macrophages (THP1-M0).

Human peripheral blood mononuclear cells (PBMCs) were freshly isolated from healthy donors via Ficoll density gradient centrifugation (P9010, Solarbio, China). CD14⁺ monocytes were further purified using magnetic-activated cell sorting (MACS) with CD14 Microbeads (130-097-052, Miltenyi Biotec, Germany) according to the manufacturer’s instructions. Purified CD14⁺ monocytes were resuspended in complete RPMI-1640 medium (RPMI-1640 supplemented with 10% FBS and 1% penicillin-streptomycin) at 1 × 10⁶ cells/mL. Human macrophage colony-stimulating factor (M-CSF, HY-P7050, MedChemExpress, USA) was added to a final concentration of 50 ng/mL. The cells were seeded into 24-well plates (1 mL/well) and differentiated for 6 days with half-volume medium changes every other day, generating PBMC-derived M0 macrophages (PBMC-M0).

### Mice

Six-week-old male BALB/c nude mice and C57BL/6 mice were obtained from Huachuang Sino Pharma Tech Co., Ltd. (Taizhou, Jiangsu, China) and housed under standard conditions. At the end of the experiments, all mice were sacrificed by CO₂ asphyxiation. All the experimental procedures were approved by the Animal Ethics Committee of the Second Affiliated Hospital of Chongqing Medical University (No. IACUC-SAHCQMU-2024-00065).

### Antibodies and reagents

The primary antibodies used for this study were as follows: anti-CD66b antibody **(**ab197678, Abcam, UK), anti-citrullinated modification of histone 3 (CitH3) antibody (ab5103, Abcam, UK), anti-neutrophil elastase (NE) antibody (89241, CST, USA), anti-MPO (Myeloperoxidase) antibody (22225-1-AP, Proteintech, China), anti-VEGF antibody (sc-57496, Santa Cruz, USA), anti-proliferating cell nuclear antigen (PCNA) antibody (BM0104, Boster, China), anti-MMP2 antibody (A23496, ABclonal, China), anti-MMP9 antibody (A25299, ABclonal, China), anti-E-cadherin antibody (A20798, ABclonal, China), anti-N-cadherin antibody (66219-1-Ig, proteintech, China), anti-CD14 antibody (11–0149-41, Thermofisher, USA), anti-CD68 antibody (ab303565, Abcam, UK), anti-CD206 antibody (24595, CST, USA), anti-CD80 antibody (M1007-10, Huabio, China), anti-IL-10 antibody (HA722032, Huabio, China), anti-IL-17 antibody (A12454, ABclonal, China), anti-p65 antibody (ET1603-12, proteintech, China), anti-p-p65(S536) antibody (HA723223, Huabio, China), anti-F4/80 antibody (ab6640, Abcam, UK), anti-CD86 antibody (30691-1-AP, proteintech, China), anti-CD31 antibody (ab182981, Abcam, UK), anti-S100A9 antibody (72590, CST, USA), **a**nti-β-actin antibody (BM0627, Boster, China). The inhibitors used for this study were as follows: IL-17R inhibitor AMG 827 (100 ng/mL; MedChemExpress, USA), DNase 1 (SLBV9316, Sigma, USA). NF-κB inhibitor BAY11-7082 (10 µM; MedChemExpress, USA), S100A9 inhibitor Paquinimod (25 µM; MedChemExpress, USA), and VEGFR inhibitor AV-951 (100 nM; MedChemExpress, USA). Recombinant protein: VEGF Protein (100 ng/mL; MedChemExpress, USA).

### Isolation of neutrophils and NETs formation assay

Neutrophils were isolated from the peripheral blood of healthy volunteers and HCC patients using the Human Peripheral Blood Neutrophil Isolation Kit (P9040, Solarbio, China), according to the manufacturer’s instructions. Briefly, neutrophil separation medium was added to 15 mL conical tubes, and then 4 mL of fresh anticoagulated whole blood was carefully layered onto the separation medium. The samples were centrifuged at 1000 × g for 40 min at room temperature. The neutrophil-rich layer was transferred to a new 15 mL conical tube. After red blood cell lysis and a series of PBS washes, the isolated neutrophils were resuspended in RPMI 1640 medium without FBS.

Purified NETs were prepared as previously described [17]. Briefly, freshly isolated neutrophils were stimulated with PMA (100 ng/mL; IP1010, Solarbio, China) and incubated at 37 °C for 4 h. The layer containing neutrophils and released NETs at the bottom was collected by centrifugation at 450 × g for 10 min at 4 °C. The NETs-rich supernatant was then collected and further centrifuged at 18,000 × g for 10 min at 4 °C. After discarding the supernatant, the pelleted NETs were resuspended in cold PBS. The concentration of NETs-DNA was measured using a spectrophotometer (ThermoFisher). For subsequent experiments, purified NETs were used at a final concentration of 2 µg/mL to treat cells.

### Immunofluorescence (IF)

For cell IF analysis, pretreated neutrophils (5 × 10^5^ cells/well) were seeded onto coverslips in 24-well plates, washed with PBS, fixed in 4% paraformaldehyde, permeabilized with 0.2% Triton X-100 (V900502, Sigma, USA) for 10 min at 37 °C, and incubated with blocking serum for 60 min at 37 °C. The slides were then incubated overnight at 4 °C with primary antibodies as follows: anti-CitH3 (1:1000), anti-NE (1:400), anti-MPO (1:400), anti-E-cadherin antibody (1:500), anti-N-cadherin antibody (1:500), anti-CD206 antibody (1:400), anti-CD80 antibody (1:200), and anti-IL-17 antibody (1:400). After washing with PBS, the cells were incubated with fluorescence-conjugated secondary antibody (1:100) and stained with DAPI (AR1177, Boster, China) for 10 min at 37 °C to visualize nuclei. The fluorescence images were acquired using a confocal fluorescence microscope.

IF staining for paraffin-embedded sections was performed similarly. The sections underwent rehydration and microwave antigen retrieval. After the elimination of autofluorescence, sections were incubated overnight at 4 °C with anti-CD66b (1:200), anti-CitH3 (1:1000), anti-CD68 (1:500), anti-CD206 (1:400), anti-F4/80 (1:400), anti-CD86 (1:300), and anti-CD206 (1:400) antibodies. Subsequently, sections were incubated with fluorescence-conjugated secondary antibodies and stained with DAPI for nuclei visualization.

### RNA isolation and quantitative real-time PCR

Total RNA was extracted from treated cells using TRIzol reagent (Invitrogen, USA) according to the manufacturer’s protocol. First-strand cDNA was synthesized from 1 µg total RNA using a Reverse Transcription kit (Takara, Japan). The mRNA levels of *VEGF*,* IL-10*,* TGF-β*,* iNOS*, and *TNF-α* were analyzed and normalized to GAPDH using a CFX96 real-time PCR detection system (Bio-Rad, USA) with SYBR Green dye (Biomake, USA) according to the manufacturer’s instructions. The primers used in this study were synthesized by Genscript (China), and the primer sequence information is shown in Table 2.

**Table 2:** The primers used in this study

Genes		Sequences
*GAPDH*	Forward primer	CAGCGACACCCACTCCTC
	Reverse primer	TGAGGTCCACCACCCTGT
*VEGF*	Forward primer	TGCAGATTATGCGGATCAAACC
	Reverse primer	TGCATTCACATTTGTTGTGCTGTAG
*IL-10*	Forward primer	CAAGACCCAGACATCAAGGCG
	Reverse primer	GCATTCTTCACCTGCTCCACG
*TGF-β*	Forward primer	CCCAGCATCTGCAAAGCTC
	Reverse primer	GTCAATGTACAGCTGCCGCA
*iNOS*	Forward primer	CAGCGGGATGACTTTCCAA
	Reverse primer	AGGCAAGATTTGGACCTGCA
*TNF-α*	Forward primer	CAGCCTCTTCTCCTTCCTGA
	Reverse primer	GGAAGACCCCTCCCAGATAGA

### Co-culture assay

For co-culture analysis in 6-well plates, THLE-2 cells (5 × 10^5^ cells/well), HepG2 cells (5 × 10^5^ cells/well), and HuH-7 cells (5 × 10^5^ cells/well) were seeded in the upper chamber of a 3 μm transwell system, while THP-1-derived macrophages (1 × 10^5^ cells/well) were seeded in the lower chamber for incubation at 24, 48, or 72 h.

### Cell proliferation assay

HuH-7 cells (2 × 10³ cells/well) and HepG2 cells (2 × 10³ cells/well) were seeded in 96-well plates and treated with conditioned media (CM) from the following groups: CM(HuH-7/HepG2), CM(HuH-7/HepG2 + M0), CM(HuH-7/HepG2 + M0 + NETs), and CM(HuH-7/HepG2 + M0 + NETs + AMG827). Cell proliferation was assessed using the Cell Counting Kit-8 (AR1160; Boster, Wuhan, Hubei, China) according to the manufacturer’s instructions. The optical density (OD) at 450 nm was measured daily for three consecutive days using a microplate reader. All experiments were performed in triplicate.

### Tube formation assay

HUVECs (2.5 × 10^4^ cells/well) were seeded onto 96-well plates precoated with Matrigel (082701; Mogengel, China) and incubated with the indicated CM. After incubation for 4 h, tube-like structures were observed and quantified under a microscope. All experiments were performed in triplicate.

### Cell migration and invasion assay

HuH-7 cells (5 × 10⁵ cells/well) and HepG2 cells (5 × 10⁵ cells/well) were seeded in the upper chamber of an 8 μm transwell system and incubated in serum-free DMEM. Various CMs supplemented with 20% FBS were added to the lower chamber of 24-well plates. After incubation for 24 h, cells that had migrated through the membrane were washed, fixed with 4% paraformaldehyde, stained with crystal violet, and observed under a microscope.

The transwell invasion assay was conducted using a similar procedure. Briefly, HuH-7 cells (1 × 10⁶ cells/well) and HepG2 cells (1 × 10⁶ cells/well) were seeded in the upper chamber of an 8 μm transwell system pre-coated with matrigel and cultured in serum-free DMEM. The subsequent steps were identical to those of the migration assay. All experiments were performed in triplicate.

### DEN-induced spontaneous model

Two-week-old C57BL/6 mice were intraperitoneally (i.p.) injected with diethylnitrosamine (DEN; 25 mg/kg; HY-N7434, MedChemExpress, Shanghai, China). Four weeks later, the mice were intraperitoneally administered carbon tetrachloride (CCl₄, 1:4, v/v in olive oil; Macklin, Shanghai, China) at a dose of 2 ml/kg body weight, twice per week, for 14 consecutive weeks. During the treatment phase, mice were randomly divided into five groups (*n* = 3/each group): the PBS group received a single intraperitoneal injection of PBS; the LPS group received a single intraperitoneal injection of lipopolysaccharide (LPS; 10 µg/mouse; L2880, Sigma, Saint Louis, USA); the LPS + DNase I group received intraperitoneal injections of lipopolysaccharide (LPS; 10 µg/mouse) and DNase I (100 U/mouse) every three days; the AMG827 group received AMG827 (2.7 mg/kg; MedChemExpress, USA) via tail vein injection every three days; and the LPS + AMG827 group received a single intraperitoneal injection of LPS followed by AMG827 tail vein injection every three days.

### Hepa 1–6 cell-derived subcutaneous model and lung metastasis model

Six-week-old BALB/c nude mice (*n* = 24) were randomly divided into four groups (*n* = 6 per group): Hepa1-6 alone, Hepa1-6 + RAW264.7, Hepa1-6 + RAW264.7 + NETs, and Hepa1-6 + RAW264.7 + NETs + AMG 827. Hepa1-6 cells (1 × 10⁶ per group) were co-cultured for 24 h either alone, with RAW264.7 cells (2 × 10⁵), with RAW264.7 plus NETs, or with RAW264.7 plus NETs and AMG 827. The mixed cells were then harvested, resuspended in 200 µL PBS, and subcutaneously injected into the left flank of each mouse. Tumor growth was monitored every three days by measuring tumor length and width with calipers, and tumor volumes were calculated using the formula:1/2×(Rmax×Rmin^2^), where R is the tumor diameter. All mice were sacrificed at day 22, and tumor tissues were harvested for further analysis.

For in vivo metastasis experiments, six-week-old male C57BL/6 mice (*n* = 3/each group) were injected via tail vein with the same cell preparations as described above (Hepa1-6: 1 × 10⁶; RAW264.7: 2 × 10⁵) under the indicated conditions. All mice were sacrificed at day 30, and lung tissues were collected for further analysis. All animal procedures were approved by the Institutional Animal Care and Use Committee.

### Immunohistochemistry (IHC) staining

Briefly, paraffin-embedded sections were rehydrated and subjected to microwave antigen retrieval, followed by blocking of endogenous peroxidase activity. Sections were then incubated overnight at 4 °C with the following primary antibodies: anti-CD31 (1:500), anti-PCNA (1:1000), anti-VEGF (1:500), anti-MMP2 (1:250), anti-MMP9 (1:500), anti-E-cadherin (1:500), anti-N-cadherin (1:500). After washing, sections were incubated for 30 min at room temperature with a peroxidase-conjugated secondary antibody (1:200). Immunoreactive signals were visualized with DAB and counterstained with hematoxylin. Images were observed under an optical microscope.

### Western blot

Briefly, cells subjected to various treatments were lysed in RIPA buffer containing protease and phosphatase inhibitors to extract total protein. Equal amounts of protein (30 µg) were separated by SDS-PAGE and transferred to PVDF membranes. The membranes were blocked with 5% BSA for 2 h, followed by incubation overnight at 4 °C with the following primary antibodies: anti-CitH3 (1:1000), anti-IL-10 (1:500), anti-VEGF (1:500), anti-IL-17 (1:800), anti-p65 (1:1000), anti-p-p65 (1:2000), anti-MMP2 (1:800), anti-MMP9 (1:1000), anti-S100A9 (1:800), and anti-β-actin (1:5000). After washing, the membranes were incubated at 37 °C for 1 h with horseradish peroxidase (HRP)-conjugated secondary antibodies (1:1000). Protein bands were visualized using ECL reagents (Millipore, Germany), and images were captured with the Bio-Rad imaging system. All experiments were performed in triplicate.

### Enzyme-linked immunosorbent assay (ELISA)

ELISA assays were performed according to the manufacturer’s instructions to assess the levels of inflammatory cytokines. The following commercial ELISA kits were used: human TGF-β (MBE0933, Mengbio, China), human VEGF (MBE0090, Mengbio, China), human IL-10 (MBE0051, Mengbio, China), human CCL13 (MBE0192, Mengbio, China) and human S100A9 (MBE0199, Mengbio, China).

The serum MPO-DNA complex was measured using a commercially available cell death detection ELISA kit (11774425001, Roche, Germany) following the manufacturer’s protocol. Briefly, 96-well plates were pre-coated with 5 µg/mL anti-MPO antibody and incubated overnight at 4 °C. After blocking with 1% BSA, samples were incubated with anti-DNA antibody for 2 h at room temperature. Plates were then washed three times with incubation buffer before adding 100 µL of peroxidase substrate, followed by incubation for 40 min in the dark. The optical density value was determined at 405 nm using a microplate reader (ST-960, KHB, China) to evaluate NETs levels. All experiments were performed in triplicate.

ELISA assay was performed to quantify neutrophil-derived NETs-IL-17. Briefly, neutrophils were isolated from peripheral blood and stimulated with inducers to generate NETs (as described previously). The concentration of NETs-DNA was determined via the PicoGreen assay and standardized to 50 ng/µL. Subsequently, the standardized NETs were digested and purified using DNase treatment to obtain NETs-related components. Finally, a commercial IL-17 ELISA kit (MK0180A, Mlbio, China) was utilized to measure NETs-IL-17 levels in the purified samples per the manufacturer’s instructions.

### Flow cytometry (FCM)

To investigate the differentiation of THP-1-derived macrophages, FCM was used to analyze the M1 marker (CD68^+^/CD86^+^) and the M2 marker (CD68^+^/CD206^+^) in different treatment groups. Briefly, harvested cells were washed with PBS and incubated at 4 °C for 30 min with FITC-conjugated mouse anti-human CD68 (333805, BioLegend, USA), PE-conjugated mouse anti-human CD86 (12–0869-42, Invitrogen, USA), or APC-conjugated mouse anti-human CD206 (321109, BioLegend, USA). After staining, cells were washed and analyzed using a FACSVantage SE flow cytometer (Becton-Dickinson, USA).

Similarly, to assess apoptotic HCC cells after treatment with CM(HuH-7/HepG2), CM (HuH-7/HepG2 + M0),CM(HuH-7/HepG2 + M0 + NETs), and CM(HuH-7/HepG2 + M0 + NETs + AMG827), a Dead Cell Apoptosis Kit (Life Technologies, USA) with Annexin V-FITC and PI was used according to the manufacturer’s instructions. All experiments were performed in triplicate.

### Whole-transcriptome sequencing analysis

Total RNA was extracted from THP-1-derived macrophages treated under different conditions, followed by mRNA isolation. The isolated mRNA was fragmented, reverse-transcribed to cDNA, and PCR-amplified. Sequencing libraries were constructed and purified using magnetic beads. Qualified libraries were sequenced on the Illumina NovaSeq 6000 platform (PE150). Raw sequencing data were processed and quality assessed using bioinformatics tools, and high-quality reads were retained for downstream analyses. Subsequently, these reads were aligned to the reference genome, and gene expression levels were quantified based on genome annotation. Differentially expressed genes (DEGs) were identified by the DESeq2 package (v1.30.1) in R (version 4.0.3). Then, Kyoto Encyclopedia of Genes and Genomes (KEGG) pathway analysis was performed using the clusterProfiler (v 4.10.1).

### Statistical analysis

Statistical analysis was performed using GraphPad software (version 10.1.2). The Kruskal-Wallis or Mann-Whitney test was used to assess statistical differences in serum MPO-DNA levels among patient groups. Correlations were analyzed using Spearman correlation analysis. Receiver operating characteristic (ROC) curves were generated to evaluate the diagnostic power of serum NETs (MPO-DNA), NETs-IL-17, VEGF and S100A9 via calculation of the area under the ROC curve (AUC), with sensitivity and specificity according to standard formulas. Differences between two groups were analyzed by Student’s t-test, while comparisons among three or more groups were performed using one-way ANOVA followed by the Newman-Keuls multiple comparison test. *p < 0.05, **p < 0.01, and ***p < 0.001 were considered significant.

## Results

### NETs levels are elevated in HCC patients, especially in advanced and metastatic stages

**Fig. 1:**
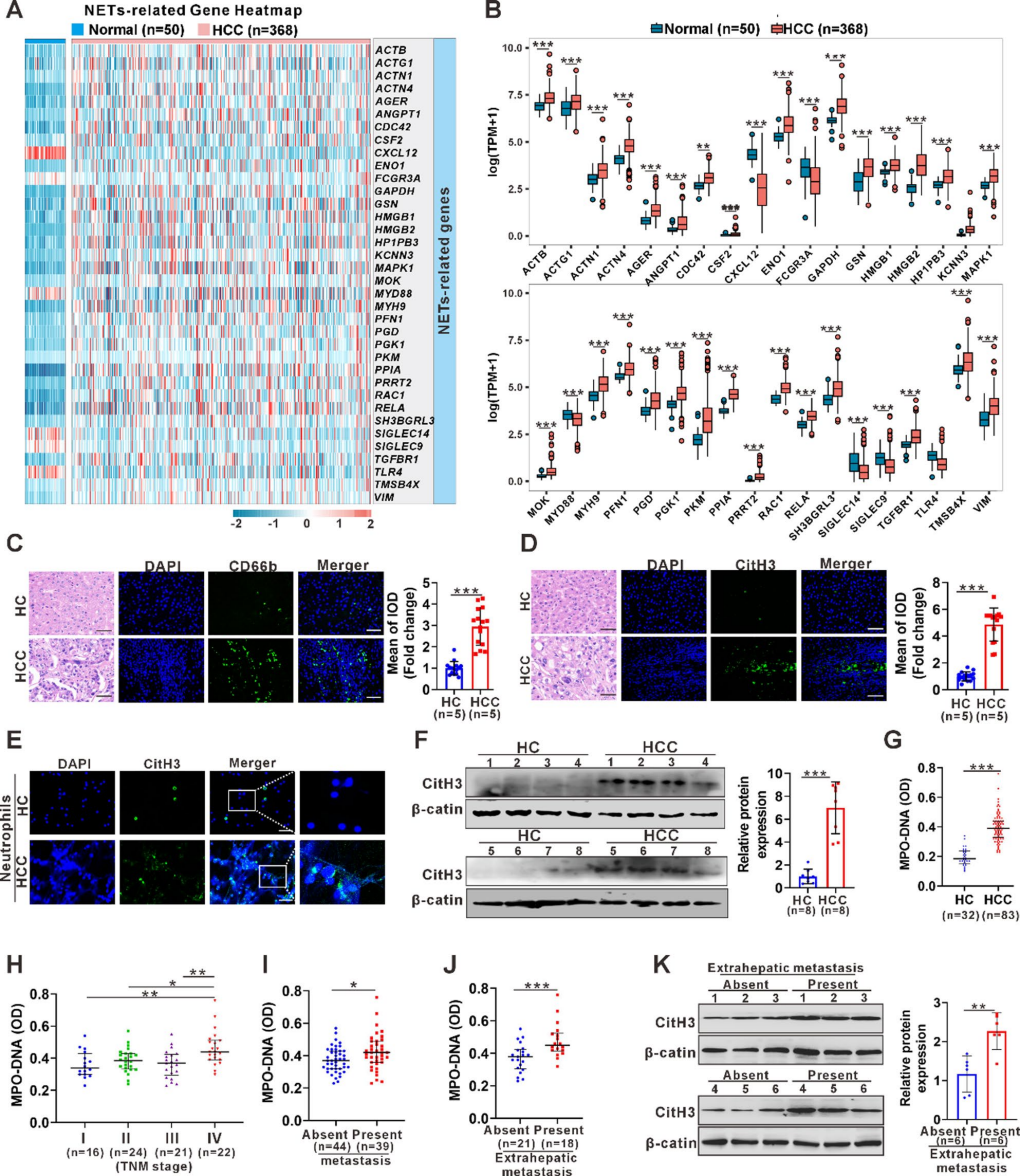
The levels of NETs increase in HCC patients, especially in advanced and metastatic stages. **A** A heatmap illustrating the differential expression patterns of NETs-related genes in normal and HCC samples based on TCGA database analysis. **B** Box plots depicting the expression differences of NETs-related genes between normal and HCC samples from the TCGA database. **C**,** D** Representative images of HE (Hematoxylin and eosin) and IF staining for CD66b (C) and CitH3 (D) in tissue sections from HCs and HCC patients. The mean IOD of CD66b or CitH3 was quantified (Image Pro Plus) from three random regions per section (HC, *n* = 5; HCC, *n* = 5) and shown as fold change relative to HCs. **E** Representative images of IF staining for CitH3 in peripheral blood neutrophils from HC and HCC patient **F** Western blot analysis of CitH3 expression in peripheral blood neutrophils from HCs (*n* = 8) and HCC (*n* = 8) patients, with band intensities normalized to β-actin and shown as fold change relative to controls **G** ELISA analysis of serum MPO-DNA levels in HCs and HCC patients. **H** ELISA analysis of serum MPO-DNA levels in HCC patients at different TNM stages (I–IV). **I** ELISA analysis of serum MPO-DNA levels in HCC patients with and without metastasis. **J** ELISA analysis of serum MPO-DNA levels in HCC patients with intrahepatic metastasis and extrahepatic metastasis. **K** Western blot analysis of CitH3 expression in peripheral blood neutrophils from HCC patients with (*n* = 6) and without (*n* = 6) extrahepatic metastasis. Band intensities were normalized to β-actin and quantified relative to the non-metastasis group White and black scale bars: 50 μm. Data are presented as median (IQR) (G-J) and other data are presented as mean ± SD. *p < 0.05, **p < 0.01, ***p < 0.001. Student’s t test (C, D, F, K), Mann–Whitney U test (B, G, I, J), Kruskal–Wallis test (H)

In the TCGA-LIHC cohort, a total of 418 samples (368 tumor tissue samples and 50 adjacent normal tissue samples) with complete clinical and transcriptomic data were available for expression analyses. Heatmap analysis of 36 NETs-related genes (Table S1) revealed that the majority were significantly upregulated in HCC tissues (Fig. 1A-B). To validate these findings in protein level, biopsies and blood specimens were collected from HCC patients and healthy controls (HCs). IF staining for CD66b was performed on randomly selected liver biopsy tissues from HCs and HCC patients to assess intrahepatic CD66b⁺ Neu infiltration. HCC tissues exhibited increased numbers of CD66b⁺ Neu compared with HCs (Fig. 1C). Consistently, IF staining for citrullinated histone H3 (CitH3), a specific NET marker, indicated elevated NET formation in HCC tissues (Fig. 1D), which was further supported by higher CitH3 expression in freshly isolated circulating neutrophils (Fig. 1E-F). Serum MPO-DNA, a circulating NET marker, was also increased in HCC patients, particularly in those with TNM stage IV disease (Fig. 1G-H). Notably, patients with metastatic HCC exhibited elevated levels, and this increase was particularly significant in those with extrahepatic metastasis (Fig. 1I-J). Elevated CitH3 expression in circulating neutrophils from HCC patients with extrahepatic metastasis was further confirmed (Fig. 1K).

### Enrichment of M2 macrophages in HCC is strongly linked to NETs formation

**Fig. 2:**
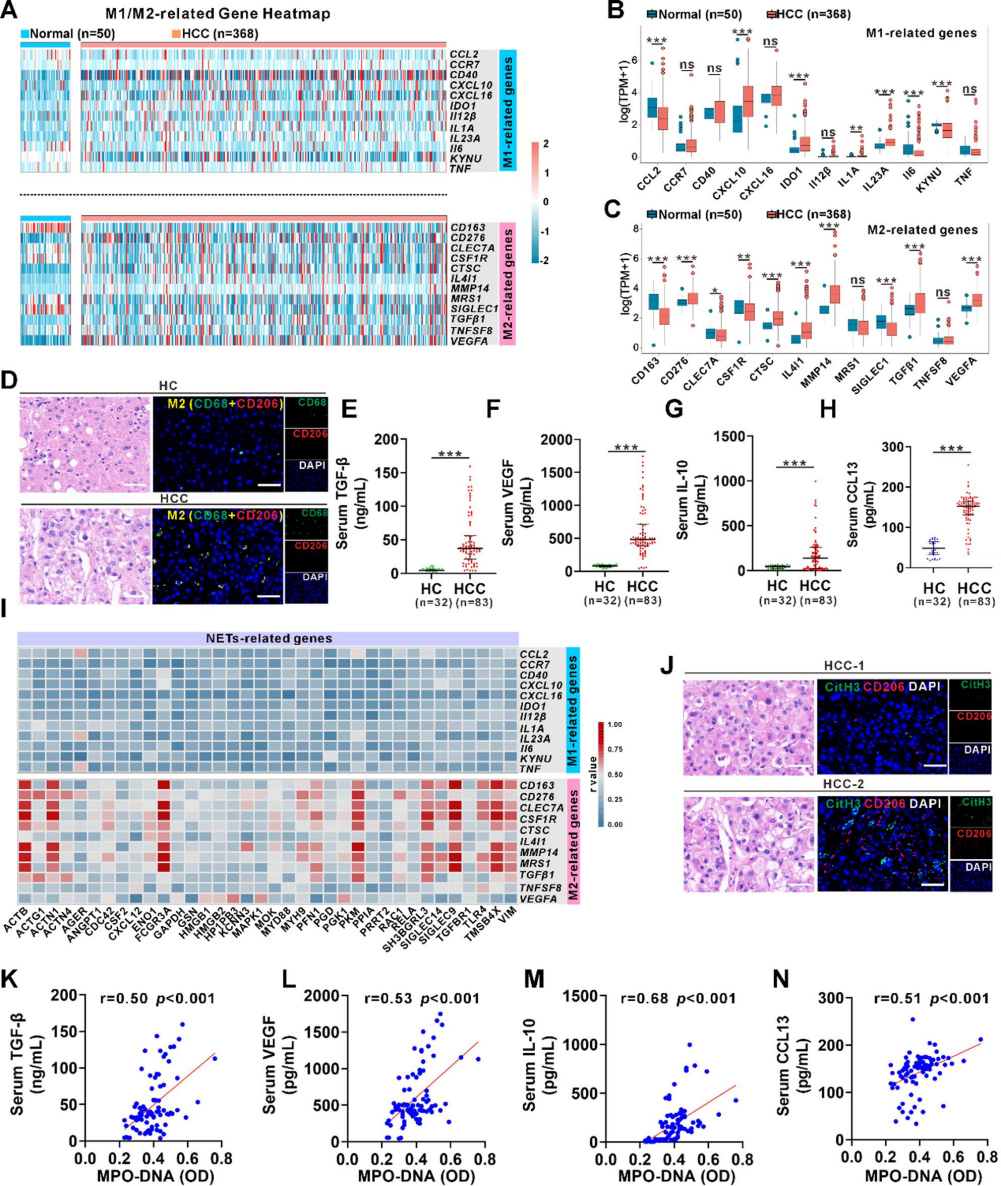
Enrichment of M2 macrophages in HCC is strongly linked to NETs formation.**A** Heatmaps illustrating the differential expression patterns of M1- and M2-related genes in normal and HCC samples based on TCGA database analysis. **B**,** C** Box plots illustrating the differential expression of M1- (**B**) and M2-related genes (**C**) in normal and HCC samples based on TCGA database analysis. **D** Representative images of HE and double IF staining for M2 macrophage markers (CD68/CD206) in tissue sections from HC and HCC patient. **E-H** ELISA analysis of serum levels of the M2d macrophage-derived cytokines TGF-β (**E**), VEGF (**F**), IL-10 (**G**), and CCL13 (**H**) in HCs (*n* = 32) and HCC patients (*n* = 83) **I** Heatmaps illustrating the correlation analysis of NETs-related genes with M1- and M2-related genes in HCC **J** Representative HE and co-IF staining images for CitH3 (NETs marker) and CD206 (M2 marker) in tissue sections from HCC patients. **K-N** Correlation analyses between serum levels of TGF-β (**K**), VEGF (**L**), IL-10 (**M**), and CCL13 (**N**) and MPO-DNA in HCC patients White scale bars: 50 μm. Data are presented as median (IQR) (E-H). ns, not significant. *p < 0.05, **p < 0.01, ***p < 0.001. All data were analyzed using the Mann–Whitney U test

Heatmap analysis of M1- and M2-related genes (Table S2) in the TCGA-LIHC cohort showed that most M2-associated genes were upregulated in HCC tissues, whereas M1-associated genes exhibited a heterogeneous expression pattern without a consistent trend (Fig. 2A-C). Using the above-mentioned specimens, we verified that a remarkable increase of CD68⁺CD206⁺ macrophages was observed in HCC tumors compared to normal liver controls by co-IF staining of CD68 and CD206 (Fig. 2D). Moreover, M2d-associated cytokines, including TGF-β, VEGF, IL-10, and CCL13, were significantly elevated in serum from HCC patients (*n* = 83) compared to HCs (*n* = 32) (Fig. 2E-H), confirming enhanced M2 macrophage accumulation in HCC patients. Our further bioinformatics correlation analysis revealed a positive association between NETs-related genes and M2 macrophage signature genes (Fig. 2I). Notably, HCC patients with increased NETs exhibited higher levels of M2 macrophage infiltration in tumor tissues (Fig. 2J), in which the NET levels were also positively correlated with serum levels of M2d-associated cytokines (Fig. 2K-N). Collectively, our data from HCC patients suggests a potential interaction between neutrophils and M2 macrophages in the TME, which prompts us to further explore their functional significance in HCC progression.

### NETs facilitate M2d macrophage polarization in the presence of HCC cells

**Fig. 3:**
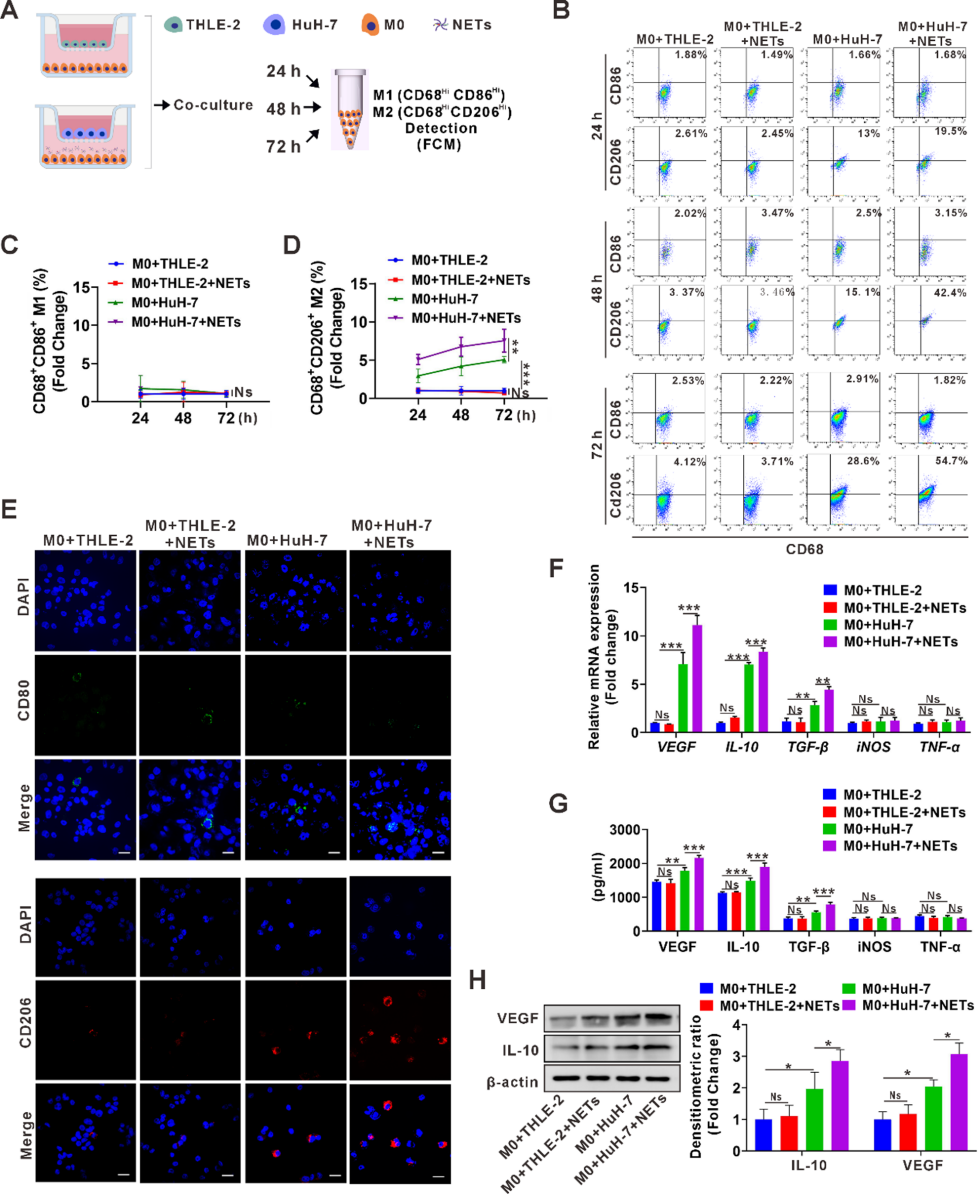
NETs facilitate M2d macrophage polarization upon co-culture with HCC cells.**A** Schematic diagram of the experimental procedure: M0 macrophages derived from THP-1 cells or human PBMCs, with or without NETs treatment, were co-cultured with normal hepatocytes (THLE-2) or HCC cells (HuH-7) for 24, 48, or 72 h. Cells were then harvested and macrophage polarization was analyzed by FCM. **B** FCM analysis of CD68^+^CD86^+^ M1 and CD68^+^CD206^+^ M2 macrophages polarized from THP-1-M0 according to procedure A. **C**,** D** Quantification of the fold change in the proportion of M1 (C) and M2 (D) polarized from THP-1-M0 from three independent experiments shown in B. **E** IF analysis of CD80^+^ M1 and CD206^+^ M2 polarized from THP-1-M0 after 48 h according to procedure A. **F** qPCR analysis of mRNA levels of M2d-related genes (*VEGF*,* IL-10*,* TGF-β*) and M1-related genes (*iNOS*,* TNF-α*) in THP-1-M0 from the above co-culture systems of A for 48 h. **G** ELISA detection of the levels of M2d-related cytokines (VEGF, IL-10, TGF-β) and M1-related cytokines (iNOS and TNF-α) in the culture supernatant from the above co-culture systems of A (THP-1-M0). **H** Western blot analysis of VEGF and IL-10 expression in THP-1-M0 from the above A co-culture systems for 48 h. Protein levels were quantified by densitometric analysis and normalized to β-actin. Data are presented as fold change compared to the control group (right panel) White scale bars: 20 μm. Data are presented as mean ± SD. Ns, not significant. *p < 0.05, **p < 0.01, ***p < 0.001. All data were analyzed using one-way ANOVA followed by the Newman-Keuls multiple comparison test

To investigate how neutrophils and macrophages interact during HCC progression, we performed NETs stimulation assays to assess their direct effects on the polarization of THP-1-derived macrophages (THP1-M0) or human PBMC-derived macrophages (PBMC-M0) (Fig. S1A). FCM analysis showed that, across the stimulation time course, neither THP-1-M0 nor PBMC-M0 displayed notable time-dependent changes in the proportions of CD68⁺CD86⁺ M1 or CD68⁺CD206⁺ M2 macrophages (Fig. S1B-C). Consistently, IF staining further confirmed these findings (Fig. S1D-E). Since NETs alone did not affect macrophage polarization, a multicellular co-culture system was established to better recapitulate the tumor microenvironment. The system consisted of THP-1-M0 or PBMC-M0 co-cultured with HCC cells or normal liver cells in the presence or absence of NETs (Fig. 3A). Co-culture with HuH-7 cells alone moderately increased the proportion of CD68⁺CD206⁺ M2 macrophages. Notably, the addition of NETs significantly enhanced M0-to-M2 polarization in a time-dependent manner, in both THP-1-M0 and PBMC-M0 (Fig. 3B-D; Fig. S2). By contrast, NETs stimulation in the presence of normal liver cells (THLE-2) did not produce any significant changes in M2 or CD68⁺CD86⁺ M1 macrophages (Fig. 3B-D, Fig. S2). IF staining data further confirmed these findings (Fig. 3E). In parallel, the mRNA levels of M2d-associated genes (*VEGF*, *IL-10*, *TGF-β*), compared to M1-associated genes (*iNOS* and *TNF-α*) were significantly upregulated in the presence of NETs and HuH-7 (Fig. 3F), which can be further verified by ELISA analysis (Fig. 3G). Western blot analysis further validated the upregulation of VEGF and IL-10 in the co-culture system of NETs and HuH-7 cells (Fig. 3H). Therefore, our data so far demonstrates that NETs could promote M2d macrophage polarization in the presence of HCC cells in vitro.

### Activation of IL-17R/NF-κB signaling by NETs-attached IL-17 is responsible for NETs-induced M2 macrophage polarization

**Fig. 4:**
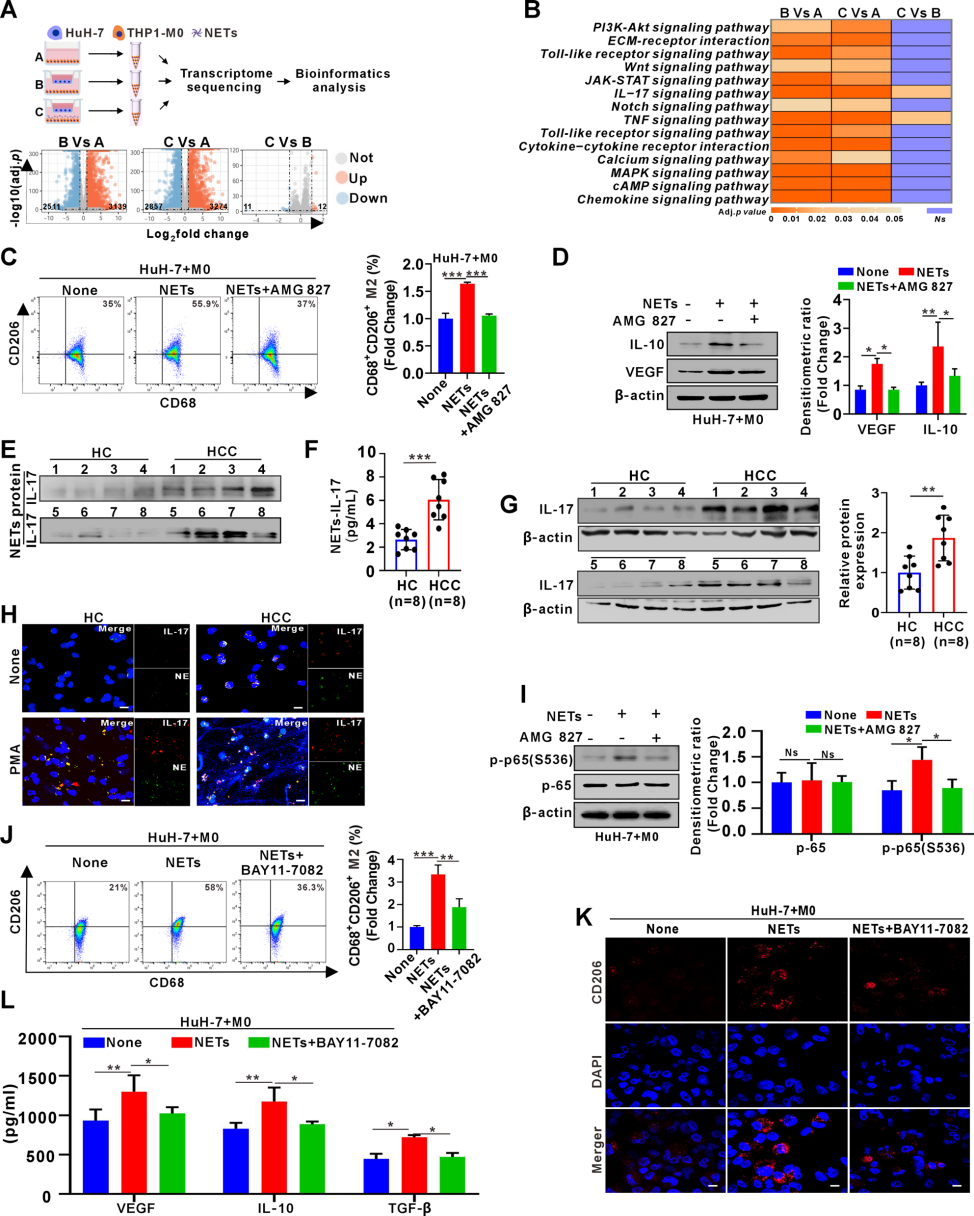
Activation of IL-17R/NF-κB signaling by NETs-attached IL-17 is responsible for NETs-induced M2 macrophage polarization.**A** Schematic illustration of the transcriptome sequencing protocol for THP1-M0 under three treatment conditions. The volcano plot shows significantly DEGs for Group B vs. A, Group C vs. A, and Group C vs. B. **B** KEGG pathway enrichment analysis of DEGs was conducted using the DAVID tool. **C** FCM analysis of the proportion of CD68^+^CD206^+^ M2 derived from THP-1-M0 following 48-hour co-culture of HuH-7 cells with NETs and (or) AMG827. Quantification of the fold change in the proportion from three independent experiments is shown in the right panel. **D** Western blot analysis of IL-10 and VEGF expression in cell lysates following the same treatments as in C. Densitometric values were normalized to β-actin and compared to the control group, with quantification shown in the right panel **E** Western blot analysis of IL-17 expression in extracted and purified NETs from peripheral blood neutrophils of HCs (*n* = 8) and HCC patients (*n* = 8). **F** ELISA analysis of IL-17 levels carried by NETs standardized to 50 ng/µL in HCs (*n* = 8) and patients with HCC (*n* = 8). **G** Western blot analysis of IL-17 expression in peripheral blood neutrophils from HCs (*n* = 8) and HCC (*n* = 8) patients. Densitometric values were normalized to β-actin and shown as a fold change relative to HCs. **H** Representative IF images of IL-17 and NE staining in peripheral blood neutrophils with (lower panel) or without PMA stimulation (upper panel) for 4 h from HCs and HCC patients. **I** Western blot analysis was conducted to assess the expression levels of p-65 and p-p65(S536) in THP-1-M0 following 48 h co-culture of HuH-7 cells with NETs and (or) AMG827. Densitometric values were normalized to β-actin and shown as fold changes relative to control in the right panel. **J** FCM was used to analyze the proportion of CD68^+^CD206^+^ M2 derived from THP-1-M0 following 48-hour co-culture of HuH-7 cells with NETs and (or) BAY11-7082. Quantification of the fold change in the proportion from three independent experiments is shown in the right panel. **K** IF analysis of CD206^+^M2 macrophage from the above co-culture systems of J treatment for 48 h. **L** ELISA analysis of secretion levels of M2d-related cytokines VEGF, IL-10 and TGF-β, in the culture supernatant from the above co-culture systems of J treatment for 48 h White scale bars: 20 μm. Data are presented as mean ± SD. Ns, not significant. *p < 0.05, **p < 0.01, ***p < 0.001. One-way ANOVA followed by the Newman-Keuls multiple comparison test (C, D, I, J, L); Student’s t test (F, G)

Next, we investigated how NETs regulate macrophage polarization. RNA-seq was performed on THP-1-derived macrophages alone (Group A), co-cultured with HuH-7 cells (Group B), or co-cultured with HuH-7 plus NETs (Group C) (Fig. 4A). DEGs (Table S3) and KEGG analysis (Table S4) showed that compared to Group A, Groups B and C commonly enriched 14 key signaling pathways, among which IL-17 and TNF signaling were highlighted in the comparison between Group C and Group B (Fig. 4B). IL-17 signaling is involved in M2 macrophage polarization [18], whereas TNF signaling mainly drives M1-like polarization [19, 20]. We then investigated whether and how IL-17 signaling may function in NETs-induced M2 macrophage polarization by introducing a common IL-17R inhibitor AMG 827. As expected, AMG827 treatment significantly restricted M2 macrophage polarization, as evidenced by a reduced proportion of CD68⁺CD206⁺ M2 macrophages and decreased expression levels of M2d-associated cytokines VEGF and IL-10 in macrophages co-cultured with NETs and HuH-7 cells (Fig. 4C-D). Consistent with the findings in THP-1-derived macrophages, human PBMC-derived macrophages showed a comparable reduction in M2 polarization under the same treatment conditions (Fig. S3). To identify the source of IL-17, we compared IL-17 protein levels in NETs isolated from HCC patients and HCs. NETs derived from HCC patients exhibited markedly higher IL-17 expression (Fig. 4E). This finding was further validated by ELISA using the same set of samples shown in Fig. 4E, which confirmed a significant increase in NET-associated IL-17 in HCC patients (Fig. 4F). Moreover, neutrophils from HCC patients also displayed substantially elevated intracellular IL-17 levels compared with those from HCs (Fig. 4G). IF staining further confirmed extensive IL-17 deposition on NETs from HCC patients (Fig. 4H), suggesting that neutrophils and NETs represent important sources of IL-17. Given that NF-κB signaling functions as a key downstream effector of IL-17R activation [21], we next investigated its role in NETs-induced M2 polarization. As expected, NETs increased NF-κB p-p65 levels in macrophages, whereas IL-17R blockade by AMG 827 reduced its upregulation (Fig. 4I). Moreover, NF-κB inhibitor BAY11-7082 decreased NETs-induced CD68⁺CD206⁺ M2 macrophages and M2d cytokines VEGF, IL-10, and TGF-β (Figs. 4J-L), indicating that NETs promote M2d polarization via IL-17R/NF-κB signaling.

### NETs-induced M2d macrophage polarization exacerbates malignant progression of HCC

**Fig. 5:**
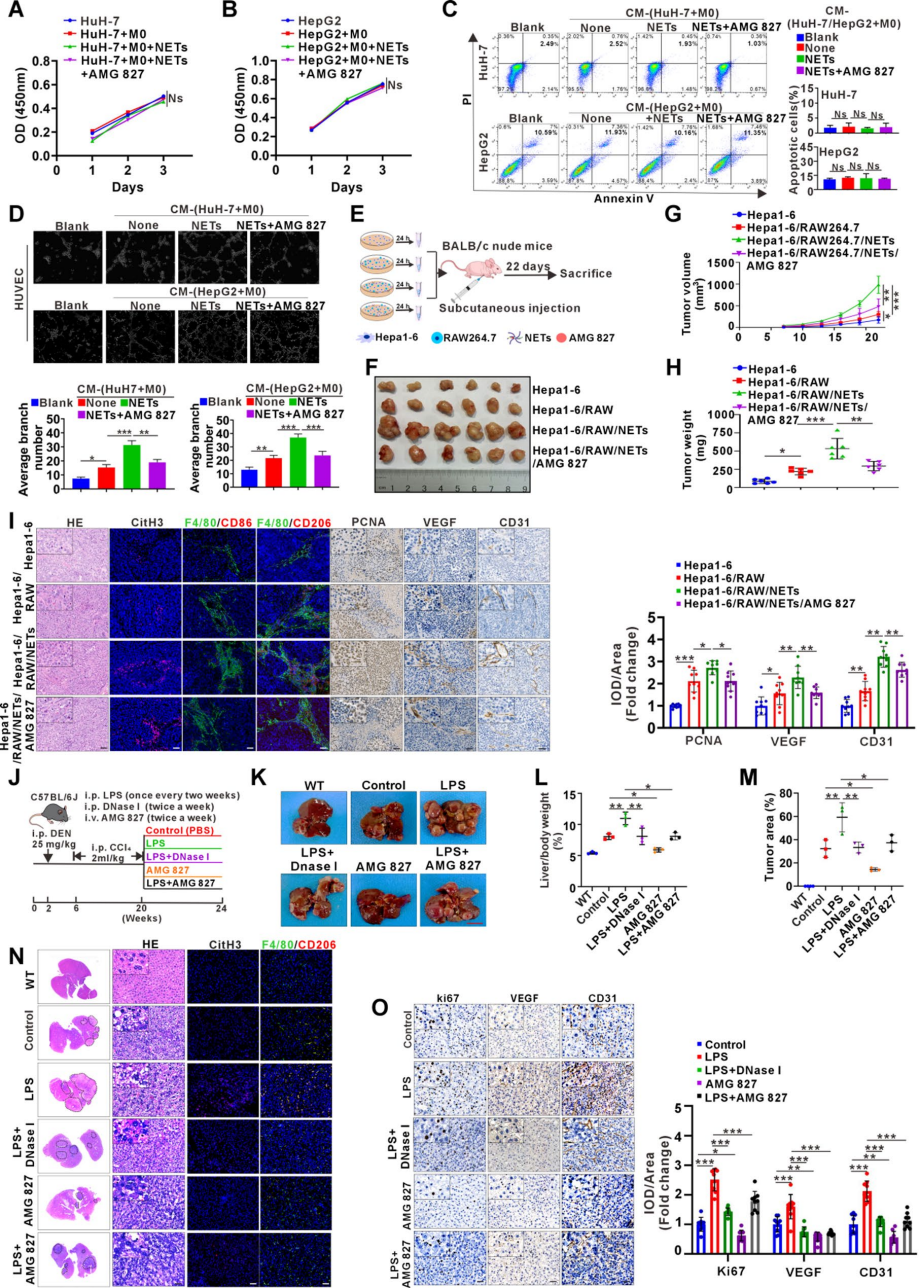
The effect of M2d macrophage polarization induced by NETs on malignant progression of HCC.**A**,** B** CCK8 assay for proliferation ability of HuH-7 (A) and HepG2 (B) cells cultured with CM(HuH-7/HepG2), CM(HuH-7/HepG2 + M0), CM(HuH-7/HepG2 + M0 + NETs), and CM(HuH-7/HepG2 + M0 + NETs + AMG827) for 24, 48, and 72 h. M0 denotes THP-1-derived M0. **C** Apoptosis analysis of HuH-7 and HepG2 cells treated with the same CM as in A-B for 48 h. Statistical data of apoptotic cells from repeated thrice. **D** Matrigel tube formation assay for HUVEC treated with CM identical to that used in C. The number of tube structures is quantified and shown below. **E** Diagram illustrating the injection method used in the subcutaneous tumor model involving nude mice. **F** Images of bearing tumors in nude mice co-injected with Hepa1-6, Hepa1-6 + RAW, Hepa1-6 + RAW + NETs and Hepa1-6 + RAW + NETs + AMG827, *n* = 6/each group. **G** Tumor growth curves for all groups. Subcutaneous tumor growth was recorded every three days with vernier calipers. **H** Graph of final tumor weights at the endpoint across all experimental groups **I** Representative images of transplanted tumor sections showing H&E staining, IF staining for CitH3, F480/CD86, and F480/CD206, and IHC staining for PCNA, VEGF and CD31. IOD values of IHC-stained sections were quantified with Image-Pro Plus and expressed as ratios relative to the Hepa 1–6 control group. Data represent mean ± SD from three randomly selected mice per group, with one section per mouse and three random fields per section. **J** Schematic diagram of the orthotopic HCC mice model induced by DEN/CCl₄. **K** Representative liver images of mice receiving different treatments. **L**,** M** Liver/body weight ratio (L) and tumor area (M) analysis from untreated WT mice and DEN/CCl₄-induced orthotopic HCC mice receiving different treatments: Control, LPS, LPS + DNase I, AMG 827, LPS + AMG827. **N**,** O** Representative images of tumor sections showing H&E staining, IF staining for CitH3, F480/CD206, and IHC staining for Ki67, VEGF and CD31. IOD values of IHC-stained sections were quantified with Image-Pro Plus and expressed as ratios relative to the Hepa 1–6 control group. Data represent mean ± SD from three randomly selected mice per group, with one section per mouse and three random fields per section White and black scale bars: 40 μm, Red scale bar: 1 cm. Data are presented as mean ± SD. Ns, not significant. *p < 0.05, **p < 0.01, ***p < 0.001. All data were analyzed using one-way ANOVA followed by the Newman-Keuls multiple comparison test

To assess the effects of NETs-mediated M2d on HCC malignancy, four CM treatments were applied: CM(HuH-7/HepG2), CM(HuH-7/HepG2 + M0), CM(HuH-7/HepG2 + M0 + NETs), and CM(HuH-7/HepG2 + M0 + NETs + AMG827). However, no significant changes in HCC proliferation or apoptosis were observed among the groups (Fig. 5A-C). As VEGF expression could be upregulated in macrophages stimulated by HuH-7 and NETs, we further studied its possible effect on angiogenesis using tube-formation assay. Indeed, CM(HuH-7/HepG2 + M0 + NETs) significantly enhanced HUVEC tube formation, which could be partially reversed by the IL-17R inhibitor AMG827 (Fig. 5D). Next, we examined the effect of NETs-mediated M2d on HCC syngeneic graft growth in vivo. Hepa1-6 cells were co-cultured with different combinations for 24 h (Hepa1-6 alone, Hepa1-6 + mouse macrophage cell line RAW264.7, Hepa1-6 + RAW264.7 + NETs, Hepa1-6 + RAW264.7 + NETs + AMG827) before transplantation (Fig. 5E). Co-injection of Hepa1-6 cells with RAW264.7 and NETs significantly increased tumor volume and weight (Fig. 5F-H). Tumor proliferation marker PCNA, angiogenesis marker CD31 and VEGF, M2 macrophage marker CD206 and NET marker CitH3 were also upregulated in parallel by IHC or IF staining, respectively, which M1 macrophage marker CD86 remained unchanged (Fig. 5I). Importantly, these effects could be partially reversed by AMG827 (Fig. 5I), verified the importance of IL-17 signaling in regulating NETs-induced M2 macrophage polarization and tumor-supportive function in HCC in vivo. To further confirm our findings, we next established a long-term spontaneous HCC model in C57BL/6J mice induced by intraperitoneal injection of DEN/CCl₄. LPS was administered to induce NETs formation, DNase I was administered intraperitoneally to suppress NET formation, and AMG827 was delivered via tail vein injection to inhibit IL-17R (Fig. 5J). The generation of NETs after LPS injection and their inhibition by DNase I were confirmed by ELISA analysis of the NETs marker MPO-DNA (Fig. S4). Again, our data generated from this spontaneous HCC mouse model demonstrated that LPS-induced NETs could significantly promote HCC progression, indicated by increased tumor burden, liver-to-body weight ratio and tumor area, which was partially reversed by AMG827 (Fig. 5K-N). Furthermore, IF/IHC staining confirmed that NETs induction increased M2 (F4/80^+^CD206^+^) accumulation as well as Ki67, CD31 and VEGF upregulation, which could be partially reversed by DNase I or AMG827 (Fig. 5N-O).

### NETs induce M2d macrophage polarization to promote HCC metastasis

**Fig. 6:**
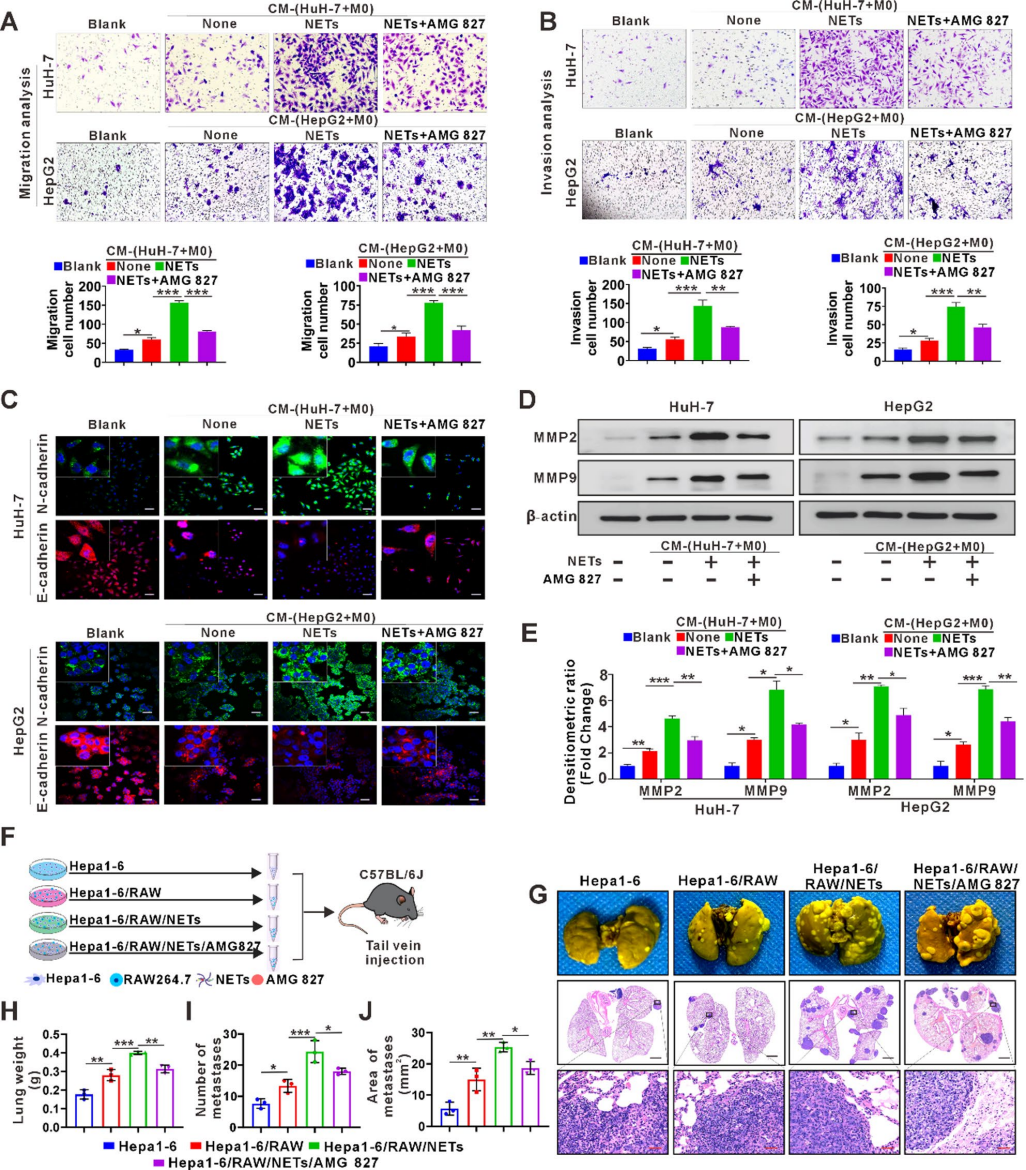
The effect of M2d macrophage polarization induced by NETs on the metastatic ability of HCC.**A** Transwell migration assay for HuH-7 and HepG2 cells exposed to various CM for 24 h: CM(HuH-7/HepG2), CM(HuH-7/HepG2 + M0), CM(HuH-7/HepG2 + M0 + NETs), and CM(HuH-7/HepG2 + M0 + NETs + AMG827). Quantitative results showing cell migration across the membrane are provided. M0 denotes THP-1-derived M0. **B** Transwell invasion assay for HuH-7 and HepG2 cells exposed to various CM identical to that used in A for 24 h. Quantitative results showing cell migration across the membrane are provided. **C** Representative IF images show E-cadherin and N-cadherin expression in HuH-7 and HepG2 cells after 24 h of treatment with various CM as in A. **D** Western blot analysis for MMP2 and MMP9 in HuH-7 and HepG2 cells after 24 h of treatment with the same conditioned media as mentioned above A. **E** Densitometric values of D were normalized to β-actin and shown as fold change relative to control groups. **F** The schematic diagram of HCC metastasis mice injected with Hepa1-6 alone, Hepa1-6 + RAW, Hepa1-6 + RAW + NETs, Hepa1-6 + RAW + NETs + AMG827. **G** Representative images of the macroscopic morphology of lungs from C57BL/6J mice with pulmonary metastases and the corresponding H&E-stained lung tissue sections. **H** Lung weight of the HCC metastasis mice model induced by Hepa1-6 cells with various treatments (*n* = 3/each group). **I** Number of metastatic foci in the HCC metastasis mouse model induced by Hepa1-6 cells with various treatments (*n* = 3/each group). **J** Metastatic area in the HCC metastasis mice model induced by Hepa1-6 cells with various treatments (*n* = 3/each group). White scale bars: 50 μm; Blank scale bars: 2 mm; Red scale bars: 100 μm. Data are presented as mean ± SD. *p < 0.05, **p < 0.01, ***p < 0.001. All data were analyzed using one-way ANOVA followed by the Newman-Keuls multiple comparison test

To investigate the effect of M2d polarization on HCC metastasis, we employed four CM treatment systems, namely CM(HuH-7/HepG2), CM(HuH-7/HepG2 + M0), CM(HuH-7/HepG2 + M0 + NETs), and CM(HuH-7/HepG2 + M0 + NETs + AMG827), which were used to treat two HCC cell lines, HuH-7 and HepG2, respectively, for analyzing metastasis-related behaviors such as cell migration and invasion. The results showed that stimulation with CM(HuH-7/HepG2 + M0 + NETs) significantly increased the number of migrated HuH-7 and HepG2 cells, whereas inhibition of macrophage polarization using CM(HuH-7/HepG2 + M0 + NETs + AMG827) partially reduced the number of migrated cells (Fig. 6A), and a similar trend was observed in the cell invasion assay (Fig. 6B). These findings indicate that NETs-mediated M2d polarization can promote the migration and invasion of HCC cells, which can be partially reversed by the IL-17R inhibitor AMG827. Further investigation of the underlying molecular mechanisms revealed that treatment with CM(HuH-7/HepG2 + M0 + NETs) decreased the expression of the epithelial marker E-cadherin and increased the expression of the mesenchymal marker N-cadherin, indicating activation of the epithelial-mesenchymal transition (EMT) process (Fig. 6C). Meanwhile, the expression levels of the invasion-related proteins MMP2 and MMP9 were also elevated in both cell lines following stimulation with CM(HuH-7/HepG2 + M0 + NETs). In contrast, treatment with CM(HuH-7/HepG2 + M0 + NETs + AMG827) reversed these molecular changes compared to the NETs-treated group (Fig. 6D-E). The changes in EMT markers and invasion-related proteins were further validated in the Hepa1-6 cell-derived subcutaneous tumor model in vivo (Fig. S5).

Next, we examined the effect of NETs-mediated M2d on HCC metastasis in vivo. Hepa1-6 cells were cocultured with different combinations for 24 h (Hepa1-6 alone; Hepa1-6 + RAW264.7; Hepa1-6 + RAW264.7 + NETs; Hepa1-6 + RAW264.7 + NETs + AMG827), and then transferred into mice via the tail vein (Fig. 6F). At day 30 post-injection, mice were sacrificed for organ collection and histopathological analysis to determine the presence of metastatic lesions in the lungs (Fig. 6G). Consistent with our in vitro data, tissue weight, numbers of metastatic nodules and metastatic area were significantly increased in the lungs from group of mice injected by Hepa1-6 + RAW264.7 + NETs. In addition, this metastasis-promoting effect can be partially reversed by addition of IL-17R inhibitor AMG827 (Fig. 6H-J).

### M2d macrophage-derived VEGF induces NETs formation by indirectly promoting HCC cell-derived S100A9

**Fig. 7:**
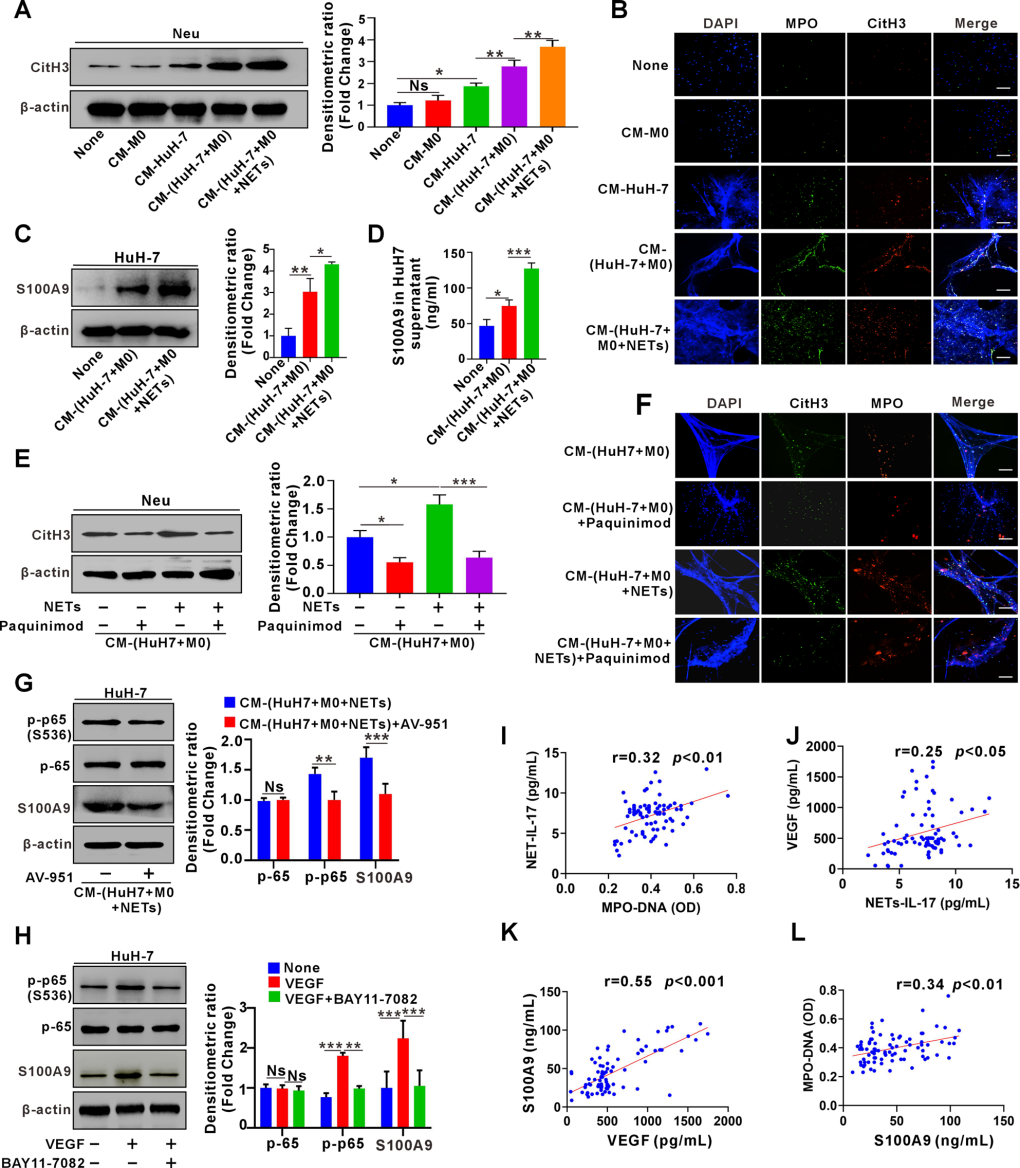
M2 macrophage-derived VEGF induces neutrophil NETs formation by indirectly promoting HCC cell-derived S100A9.**A** Western blot analysis for CitH3 expression in neutrophils treated with and without CM-M0, CM-HuH-7, CM-(HuH-7 + M0), or CM-(HuH-7 + M0 + NETs) for 12 h. Densitometric values were normalized to β-actin and compared to the control group, with quantification shown in the right panel. M0 denotes THP-1-derived M0. **B** Representative IF images of DNA/MPO/CitH3 staining in neutrophils subjected to the same treatments as shown in A. **C** Western blot analysis for S100A9 expression in HuH-7 cells treated with CM-(HuH-7 + M0) or CM-(HuH-7 + M0 + NETs) for 24 h. Densitometric values were normalized to β-actin and compared to the control group, with quantification shown in the right panel. M0 denotes THP-1-derived M0. **D** ELISA analysis of S100A9 levels in the culture supernatant of HuH-7 cells, using the same grouping as described in C. **E** Western blot analysis of CitH3 expression in neutrophils following 12 h treatment with CM-(HuH-7-M0) or CM-(HuH-7-M0 + NETs), with or without Paquinimod. Densitometric values were normalized to β-actin and compared to the control group, with quantification shown in the right panel. M0 denotes THP-1-derived M0. **F** Representative IF images of DNA/MPO/CitH3 staining in neutrophils subjected to the same treatments as described in E. **G** Western blot analysis for S100A9, p-p65(S536), and p65 expression in HuH-7 cells treated with CM-(HuH-7-M0 + NETs), with or without AV-951 for 24 h. Densitometric values were normalized to β-actin and compared to the control group, with quantification presented in the right panel. M0 denotes THP-1-derived M0. **H** Western blot analysis of S100A9, p-p65(S536), and p65 expression in HuH-7 cells treated with or without exogenous VEGF, with or without BAY11-7082 for 24 h. Densitometric values were normalized to β-actin and compared with the control group, with quantification shown in the right panel. **I** Correlation analysis between serum MPO-DNA levels and NETs-IL-17 levels in HCC patients. **J** Correlation analysis between NETs-IL-17 levels and serum levels of VEGF in HCC patients. **K** Correlation analyses between serum levels of VEGF and S100A9 in HCC patients. **L** Correlation analyses between serum levels of S100A9 and MPO-DNA in HCC patients White scale bars: 30 μm. Data are presented as mean ± SD. Ns, not significant. *p < 0.05, **p < 0.01, ***p < 0.001. One-way ANOVA followed by the Newman-Keuls multiple comparison test (A, C, D, E, H); Student’s t test (G)

We next investigated whether NETs-induced M2d, in turn, regulate NETs release from neutrophils. Neutrophils were exposed to CM from M0 macrophages (CM-M0), CM from HuH-7 cells (CM-HuH-7), and CM from HuH-7/M0 co-cultures either lacking NETs (CM-(HuH-7 + M0)) or supplemented with NETs (CM-(HuH-7 + M0 + NETs)). CM-M0 did not significantly affect CitH3 (a NET marker) expression. By contrast, CM-HuH-7 induced CitH3 upregulation, which was further enhanced by CM-(HuH-7 + M0), and reached its highest level with CM-(HuH-7 + M0 + NETs) (Fig. 7A). Similar observations were confirmed by IF analysis for NETs marker (DNA/MPO/CitH3) detection (Fig. 7B). These results indicate that factors derived from HuH-7 cells facilitate NET formation, and that crosstalk with NETs-induced M2d exerts an even greater stimulatory effect. Since our previous work demonstrated that S100A9 released from HCC cells facilitates NET formation [6], we next investigated whether the enhanced NET formation observed here was associated with altered S100A9 expression. Indeed, exposure of HuH-7 cells to CM-(HuH-7 + M0 + NETs) markedly upregulated S100A9 expression (Fig. 7C), a finding further validated by ELISA (Fig. 7D). To clarify the involvement of S100A9, we added a specific S100A9 inhibitor, Paquinimod, in the above CM. Addition of Paquinimod in CM-(HuH-7 + M0) or CM-(HuH-7 + M0 + NETs) significantly downregulated CitH3 levels in neutrophils (Fig. 7E), which was also confirmed by IF analysis (Fig. 7F), indicating that NETs-induced M2d promote NETs formation by inducing S100A9 expression in HCC cells.

We next explored M2d-derived factors regulating S100A9 in HCC cells. Since NF-κB drives S100A9 expression [22] and M2d secrete abundant VEGF, which activates NF-κB and correlates with S100A9 upregulation [23–26], we hypothesized that M2d induce S100A9 via the VEGF–NF-κB signaling axis. To test this, HuH-7 cells were treated with CM-(HuH-7 + M0 + NETs) in the presence or absence of the VEGFR inhibitor AV-951. VEGFR blockade significantly reduced both p-p65(S536) and S100A9 levels in HuH-7 cells (Fig. 7G). Notably, treatment with AV-951 alone had no effect on S100A9 expression, excluding any direct effect of the inhibitor itself (Fig. S6). To further validate this pathway, HuH-7 cells were stimulated with exogenous VEGF. Exogenous VEGF increased S100A9 and p-p65 levels, whereas co-treatment with the NF-κB inhibitor BAY11-7082 abolished these effects (Fig. 7H). To elucidate the regulatory interactions among NETs-IL-17, MPO-DNA, VEGF, and S100A9 in HCC, we systematically analyzed the correlations in their secretion and expression levels. First, neutrophil-derived NETs-IL-17 levels showed a significant positive correlation with the serum NETs marker MPO-DNA (Fig. 7I). Building on this, we observed that VEGF, a cytokine associated with M2d macrophages, was also positively correlated with NETs-IL-17, suggesting that NETs-IL-17 may promote VEGF secretion in M2d macrophages (Fig. 7J). Further analysis revealed a positive correlation between S100A9 and VEGF, indicating that VEGF may enhance S100A9 expression in HCC cells (Fig. 7K). In addition, S100A9 was positively correlated with MPO-DNA, suggesting the existence of a feedback loop in which S100A9 may further stimulate NETs formation by neutrophils (Fig. 7L).

### The clinical significance of neutrophil-derived NETs-IL-17 in HCC patients

**Fig. 8:**
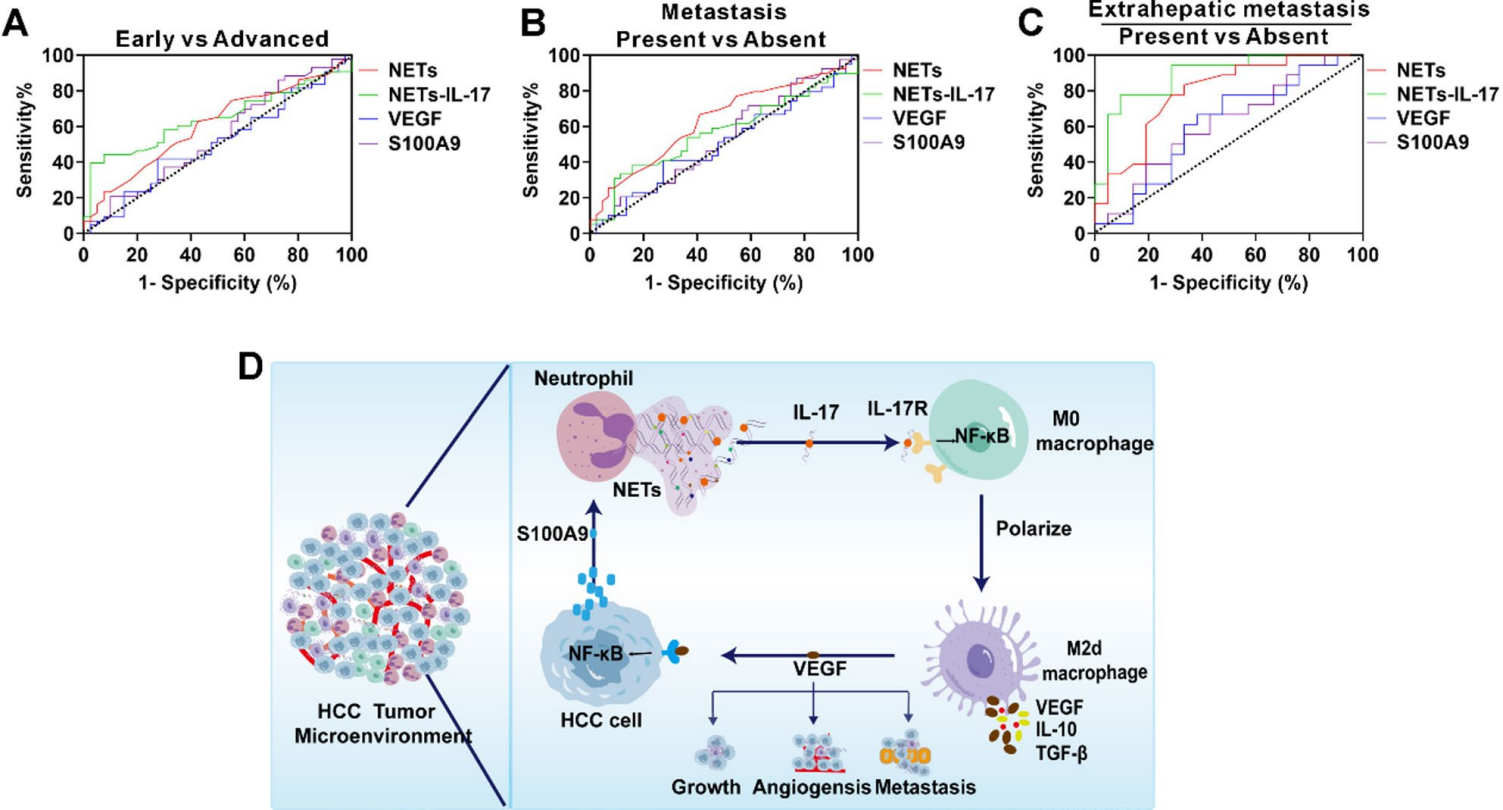
The clinical significance of NETs-IL-17 in HCC patients.**A** ROC curve of serum NETs, NETs-IL-17, serum VEGF and serum S100A9 for identifying advanced stage from early stage in HCC patients. **B** ROC curve of serum NETs, NETs-IL-17, serum VEGF and serum S100A9 for predicting metastasis in HCC patients. **C** ROC curve of serum NETs, NETs-IL-17, serum VEGF and serum S100A9 for predicting extrahepatic metastasis in HCC patients. **D** Schematic illustration of the proposed working model: IL-17 carried by NETs activates the IL-17/NF-κB signaling pathway, promoting the polarization of M2d macrophages. These polarized M2d macrophages secrete VEGF, which stimulates HCC cells to produce S100A9. S100A9, in turn, induces neutrophils to release additional NETs, thereby establishing a positive feedback loop

We evaluated the distribution of neutrophil-derived NETs-IL-17 across different clinicopathological subgroups (Table 3). Stratification analyses based on sex, age, tumor size, and tumor number showed no significant differences in NETs-IL-17 levels, indicating that this biomarker is not affected by these baseline clinical variables. We next assessed the diagnostic relevance of circulating NETs, NETs-IL-17, VEGF, and S100A9 using ROC curve analyses. For differentiating advanced-stage from early-stage HCC, these biomarkers exhibited limited discriminative ability, with AUCs of 0.61, 0.65, 0.51, and 0.54, respectively (Fig. 8A). A similarly modest performance was observed for predicting overall metastatic status, yielding AUCs of 0.64, 0.57, 0.51, and 0.53, respectively (Fig. 8B). Notably, the predictive value improved markedly when specifically evaluating extrahepatic metastasis. The AUCs for circulating NETs and NETs-IL-17 increased to 0.80 and 0.89, respectively, both significantly outperforming VEGF (0.61) and S100A9 (0.62) (Fig. 8C). Among allbiomarkers examined, NETs-IL-17 displayed the highest ability to identify extrahepatic metastasis.

**Table 3:** Distribution of NETs-IL-17 in HCC patients with various clinicopathological parameters

Parameter	HCC (*n* = 83)		
	*n* (%)	NETs-IL-17	*p* value
Gender			
Male n (%)	54 (65.06)	7.60 (2.65)	0.83
Female n (%)	29 (34.94)	7.43 (3.17)	
Age (years)			
< 60 n (%)	52 (62.65)	7.60 (2.19)	0.46
≥ 60 n (%)	31 (37.35)	7.44 (2.98)	
Tumor diameter			
< 50 mm n (%)	67 (80.72)	7.53 (2.75)	0.72
≥ 50 mm n (%)	16 (19.28)	7.64 (2.51)	
Tumor number			
Solitary n (%)	57 (68.67)	7.43 (2.35)	0.50
Multiple n (%)	26 (31.33)	7.60 (3.13)	
TNM stage			
I + II n (%)	40 (48.19)	7.39 (2.50)	0.06
III + IV n (%)	43 (51.80)	7.91 (2.53)	
Metastasis			
Absent n (%)	44 (53.01)	7.43 (2.52)	0.52
Present n (%)	39 (46.99)	7.65 (2.80)	
Extrahepatic metastasis status in total metastatic cases			
Absent n (%)	21 (53.84)	6.54 (3.48)	0.0001
Present n (%)	18 (46.16)	8.47 (1.80)	

NETs-IL-17, with NETs standardized to 50 ng/mL, are presented as median (interquartile range). *p* value < 0.05 are considered as significant. *n* Number of samples, *HCC* Hepatocellular Carcinoma

## Discussion

TAMs orchestrate chronic inflammation and immune evasion in HCC, yet their interplay remains unclear. Here, we identified a feedback loop in which neutrophil-derived NETs induce macrophage polarization to the M2d phenotype via IL-17/IL-17R–NF-κB signaling. M2d macrophages release VEGF, stimulating HCC cells to secrete S100A9, which further promotes NET formation. This self-sustaining neutrophil-macrophage crosstalk accelerates tumor growth and metastasis, highlighting the NETs–IL-17/VEGF/S100A9 axis as a potential therapeutic target (Fig. 8D).

NETs are key mediators in the tumor microenvironment, where they both trap tumor cells and actively regulate immune responses [4, 27]. Previous studies have highlighted NETs’ immunosuppressive effects, particularly on CD8⁺ T cells and NK cells, by sequestering tumor cells, inducing T cell exhaustion via inhibitory checkpoints and PD-L1, and reducing effector cytokine secretion [28, 29]. Beyond adaptive immunity, NETs can be captured and degraded by macrophages [30], induce macrophage pyroptosis, as we previously observed in models of hepatitis and liver fibrosis [31] and actively modulate M1 macrophage polarization in gouty inflammation [32], highlighting that NETs also interact with macrophages and can differentially regulate their phenotypes depending on the microenvironment. In this study, we show that in HCC, NETs preferentially reprogram macrophages toward the pro-tumorigenic M2d phenotype through IL-17/IL-17R–NF-κB signaling. This polarization is consistent with previous findings that IL-17–induced M2-like macrophages, characterized by upregulation of CD206 and CD163 and increased secretion of immunosuppressive cytokines such as IL-10, TGF-β, and VEGF [18]. These results reveal a novel role for NETs as a bridge between neutrophils and macrophages, reinforcing innate immune circuits that sustain tumor growth and metastasis, and provide mechanistic insight into how innate immune cell crosstalk contributes to HCC progression.

M2 macrophages are highly plastic within the TME and can be divided into subsets, among which M2d represents a pro-tumorigenic axis closely related to classical TAM2. In HCC, M2d macrophages have been reported to promote tumor progression by facilitating angiogenesis, enhancing epithelial-mesenchymal transition, and supporting metastasis, highlighting their critical role in shaping an immunosuppressive microenvironment [33]. Here, although NETs potently promote M2d polarization in the HCC microenvironment, they do not directly induce macrophage differentiation on their own, indicating that NETs require tumor-derived signals to exert their full effect. Notably, these results clarify that the promotional effect of NETs on M2 macrophage polarization is not independent but highly dependent on the TME orchestrated by crosstalk between macrophages and HCC cells. This interdependency underscores the essential role of cell-cell interactions in shaping the functional outcome of NETs. In addition, HCC cells alone can also induce M2d macrophage polarization to a lesser extent, which may be attributable to the previously reported high levels of stimulatory factors, such as IL-6, secreted by HCC cells [34]. Similar to the functional heterogeneity exhibited by neutrophils accumulating in the TME, TAMs are highly diverse, and understanding the roles of M2 subsets may allow selective targeting of pro-tumorigenic M2d while sparing macrophages that support tissue homeostasis or antitumor immunity, thereby informing precision immunotherapy strategies for liver cancer.

A particularly novel aspect of this study is the VEGF–S100A9 axis that sustains reciprocal interactions among neutrophils, macrophages, and tumor cells. VEGF is well known for angiogenesis and as a therapeutic target in HCC [35], it also modulates immunity by inhibiting CD8⁺ T cell infiltration [36], impairing dendritic cell maturation [37], and promoting accumulation of regulatory T cells [38] and myeloid-derived suppressor cells [39]. We show that NETs-induced M2d macrophages secrete VEGF, which stimulates HCC cells to produce S100A9, a DAMP that promotes NET formation via ROS-dependent TLR4/RAGE signaling [6]. Tumor-derived S100A9 feeds back to neutrophils, completing a self-reinforcing pro-tumorigenic circuit. This VEGF–S100A9 axis highlights how VEGF extends beyond angiogenesis to orchestrate immunomodulatory loops and positions S100A9 as both a biomarker and potential therapeutic target, providing a mechanistic explanation for the chronic inflammatory and immunosuppressive niche driving HCC growth and metastasis.

The identification of the NETs–IL-17/VEGF/S100A9 axis has important biological and clinical implications. Mechanistically, it explains how neutrophils and macrophages establish a self-sustaining inflammatory circuit that drives HCC progression, highlighting their reciprocal crosstalk in perpetuating immunosuppression and chronic inflammation. Clinically, elevated circulating NETs [6], IL-17 [40], VEGF [35] and S100A9 [22, 41, 42] correlate with advanced disease, vascular invasion, and poor prognosis. Therapeutically, multiple nodes of this feedback loop are druggable: NETs formation can be disrupted by PAD4 inhibitors or DNase I treatment, both of which have shown efficacy in preclinical cancer models [27]; IL-17/IL-17R signaling can be targeted with monoclonal antibodies, already in clinical use for autoimmune diseases [43]; VEGF blockade with Ramucirumab or multikinase inhibitors (sorafenib, lenvatinib) is already standard-of-care in HCC [35], and our study suggests that these agents may also attenuate the neutrophil–macrophage feedback; S100A9 inhibitors (e.g., tasquinimod) are under clinical investigation and could potentially synergize with VEGF or IL-17 blockade to disrupt this vicious cycle. Furthermore, given that HCC often exhibits poor response to immune checkpoint inhibitors (ICIs), likely due to its profoundly immunosuppressive microenvironment [44]. Targeting this axis may also reprogram the immunosuppressive TME to enhance response to ICIs, as high neutrophil or S100A9 signatures are associated with reduced PD-1/PD-L1 efficacy [41, 45]. Together, these findings provide a rationale for combination strategies to disrupt the pro-tumorigenic feedback loop in HCC.

Despite these novel insights, several limitations exist. Our clinical validation involved a small HCC cohort, and larger multicenter studies are needed to confirm the prognostic value of the NETs–IL-17/VEGF/S100A9 axis. Mechanistic analyses were mainly in vitro and in murine models, which may not fully capture human HCC complexity. Other immune populations, such as regulatory T cells and CD8⁺ T cells, may also modulate this axis. Future studies should explore this feedback loop across different HCC etiologies using single-cell RNA sequencing, spatial transcriptomics, and multiplex IF. Preclinical testing of NETs, IL-17R, VEGF, or S100A9 inhibitors, alone or with immune checkpoint blockade, will be important to assess translational potential and guide therapies that suppress tumor progression while reprogramming the HCC microenvironment toward effective antitumor immunity.

Our findings highlight the mechanistic and translational significance of the neutrophil-macrophage feedback loop. While NETs-IL-17 has limited value for distinguishing advanced HCC stages, it shows markedly superior performance in detecting extrahepatic metastasis. Conventional markers such as circulating tumor cells (CTCs) are limited by low abundance, methodological variability, and inability to capture immune and microenvironmental processes [46]. In contrast, neutrophil-derived NETs-IL-17 reflects functional neutrophil activation and metastatic niche remodeling, making it a promising non-invasive biomarker for early detection of high-risk patients and guiding personalized surveillance and therapy.

In conclusion, the present findings reveal a positive feedback loop in the HCC tumor microenvironment, mediated by crosstalk between neutrophils and macrophages via the NETs-IL-17/VEGF/S100A9 axis. Specifically, NETs accumulation promotes M2d macrophage polarization via IL-17R/NF-κB signaling, and in turn, M2d macrophages enhance NETs formation through VEGF-induced upregulation of HCC cell-derived S100A9. This reciprocal interaction accelerates HCC progression and metastasis, highlighting the NETs-macrophage axis as a potential target for therapeutic intervention and prognostic evaluation. Importantly, these results further suggest that NETs-IL-17 may serve as a non-invasive biomarker for predicting extrahepatic metastasis in HCC.

## Supplementary Information

Supplementary Material 1

Supplementary Material 2

## Data Availability

The data that support the findings of this study are available from the corresponding author upon reasonable request.

## Abbreviations

CitH3: Citrullinated histone H3
CM: Conditioned media
CTCs: Circulating tumor cells
DAMPs: Damage-associated molecular patterns
DEN: Diethylnitrosamine
ELISA: Enzyme-linked immunosorbent assay
EMT: Epithelial–mesenchymal transition
FCM: Flow Cytometry
FBS: Fetal bovine serum
HCC: Hepatocellular carcinoma
HCs: Healthy controls
HE: Hematoxylin and eosin
HRP: Horseradish peroxidase
ICIs: Immune checkpoint inhibitors
IF: Immunofluorescence
IHC: Immunohistochemistry
LPS: Iipopolysaccharide
MACS: Magnetic-activated cell sorting
MPO: Myeloperoxidase
M-CSF: Macrophage colony-stimulating factor
NE: Neutrophil elastase
NETs: Neutrophil extracellular traps
OD: Optical density
PBMCs: Peripheral blood mononuclear cells
PCNA: Proliferating cell nuclear antigen
PMA: Phorbol 12-myristate 13-acetate
ROC: Receiver operating characteristic
TAMs: Tumor-associated macrophages
TME: Tumor microenvironment
