# Supplementary material for: Neutrophil-macrophage crosstalk via NETs–IL-17/VEGF/S100A9 axis promotes hepatocellular carcinoma progression
Source: J Exp Clin Cancer Res. 2025 Dec 30;45:27. doi: 10.1186/s13046-025-03618-x (PMC12853889; doi:10.1186/s13046-025-03618-x)
Supplement: Supplementary file 2 — Supplementary Material 2 [file 13046_2025_3618_MOESM2_ESM.docx]

**
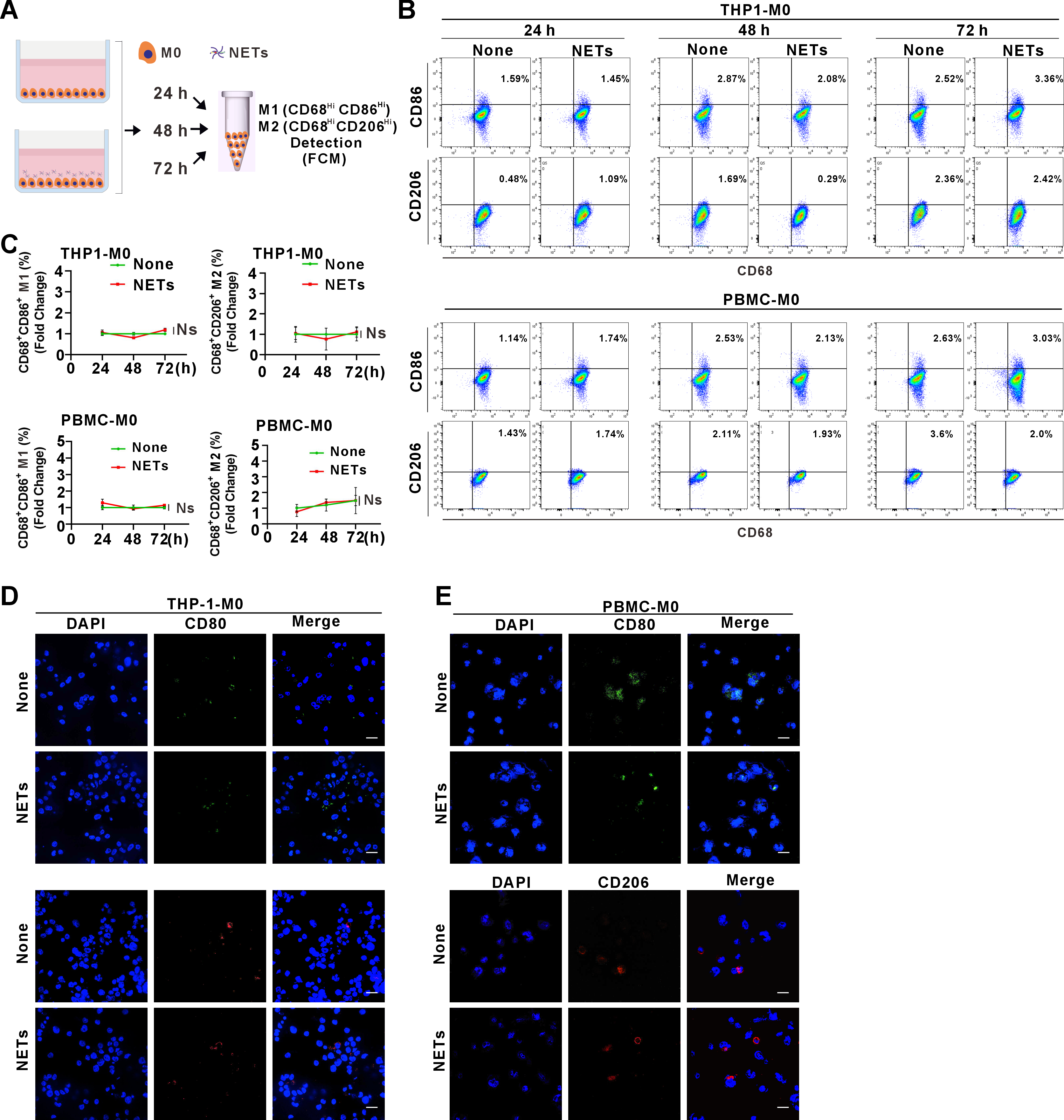
Fig. S1 A** Schematic diagram of the experimental procedure: THP-1-derived or human PBMC-derived M0 was treated with or without NETs for 24, 48, or 72 hours. Cells were then harvested, and macrophage polarization was analyzed by FCM.

**B** FCM analysis of CD68^+^CD86^+^ M1 and CD68^+^CD206^+^ M2 macrophages derived from THP-1-M0 or PBMC-M0 according to procedure A.

**C** Quantification of the fold change in the proportion of M1 and M2 macrophages from three independent experiments shown in B.

**D, E** IF analysis of CD80⁺ M1 and CD206⁺ M2 macrophages derived from THP1-M0 (D) or PBMC-M0 (E), with or without NET treatment for 48 h.

White scale bars: 20 μm. Data are presented as mean ± SD. Ns, not significant. All data were analyzed using Student’s t test.


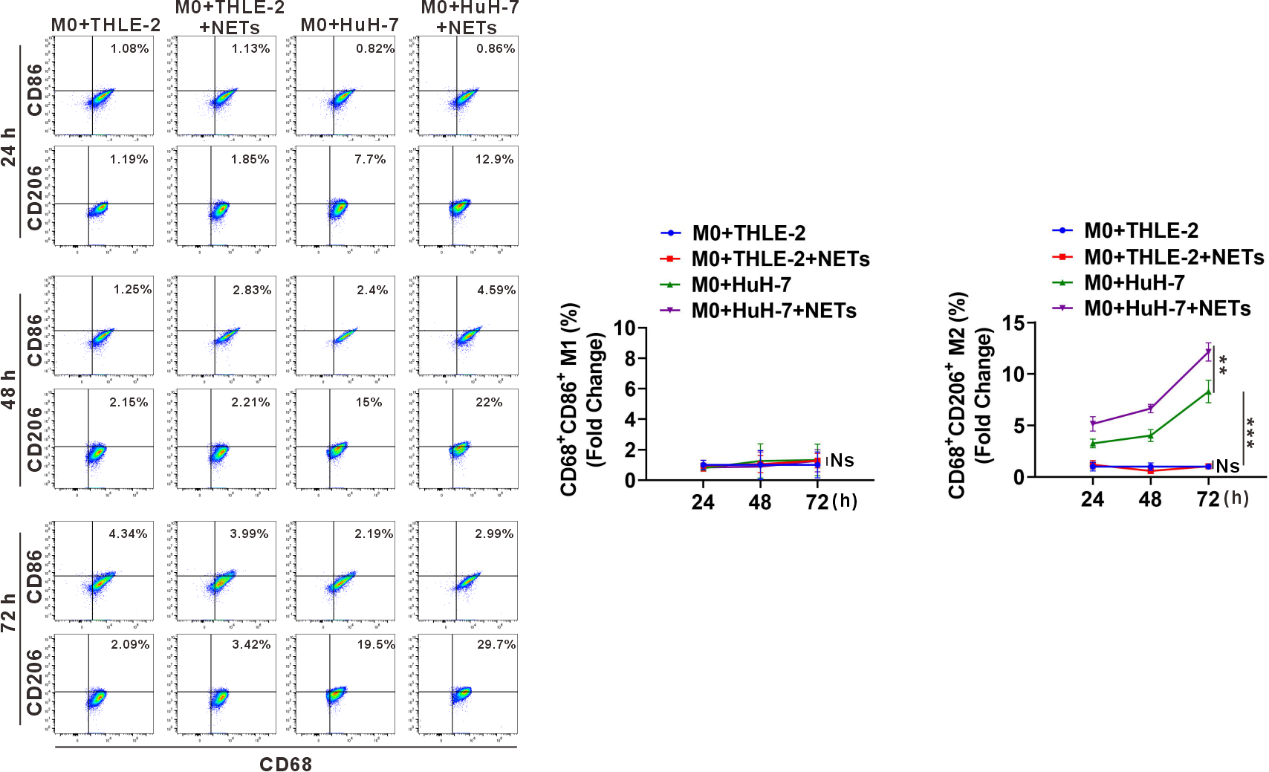


**Fig. S2** FCM analysis of CD68^+^CD86^+^ M1 and CD68^+^CD206^+^ M2 derived from PBMC-M0 according to procedure A (Fig.3A**)**. Quantification of the fold change in the proportion of M1 and M2 macrophages from three independent experiments shown in the right figure.

Data are presented as mean ± SD. Ns, not significant. ***p* < 0.01, ****p* < 0.001. All data were analyzed using one-way ANOVA followed by the Newman-Keuls multiple comparison test.

**
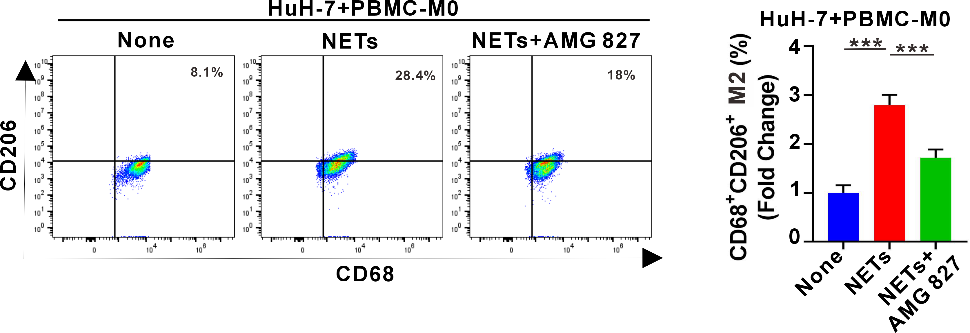
**

**Fig. S3** FCM analysis of the proportion of CD68^+^CD206^+^ macrophages derived from PBMC-M0 following 48 h co-culture of HuH-7 cells with NETs and (or) AMG827. Quantification of the fold change in the proportion from three independent experiments is shown in the right panel.

Data are presented as mean ± SD. ****p* < 0.001. All data were analyzed using one-way ANOVA followed by the Newman-Keuls multiple comparison test.


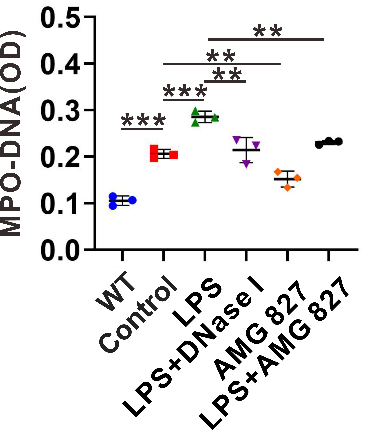


Fig. S4 ELISA analysis of serum MPO-DNA levels from untreated WT mice and DEN/CCl₄-induced orthotopic HCC mice subjected to different treatments: Control, LPS, LPS + DNase I, AMG 827, and LPS + AMG 827. Data are presented as mean ± SD. ***p* < 0.01, ****p* < 0.001. All data were analyzed using one-way ANOVA followed by the Newman-Keuls multiple comparison test.


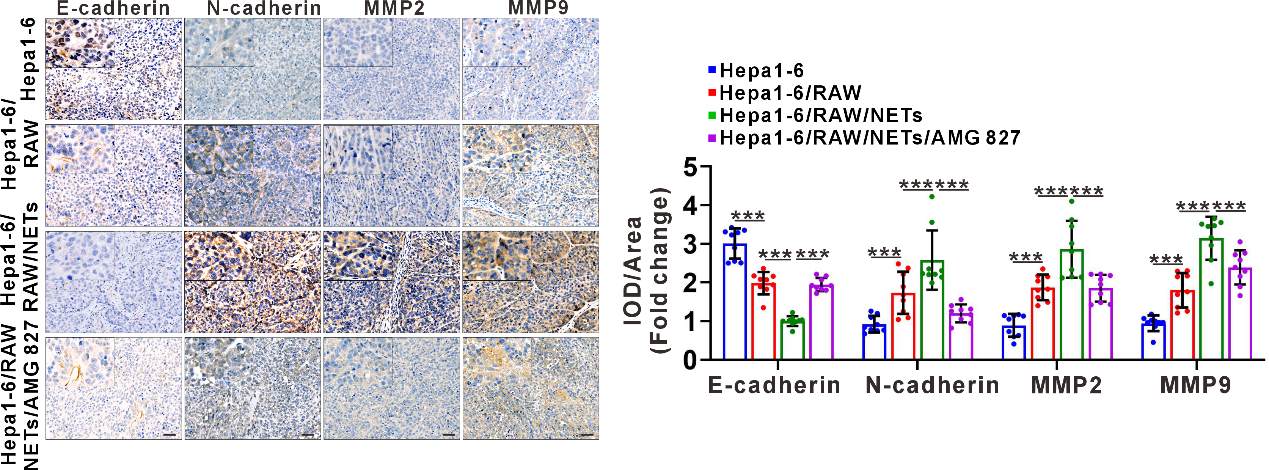


Fig. S5 Representative images of transplanted tumor sections exhibiting IHC staining for E-cadherin, N-cadherin, MMP2, and MMP9 are shown. The IOD of IHC-stained sections was quantified using Image-Pro Plus software. Quantitative data are presented as the mean ± SD from three randomly selected mice per experimental group, with one tissue section analyzed per mouse and three randomly selected fields evaluated per section. Black scale bars: 40 μm. Data are presented as mean ± SD.****p* < 0.001. All data were analyzed by one-way ANOVA, followed by the Newman-Keuls multiple comparison test.


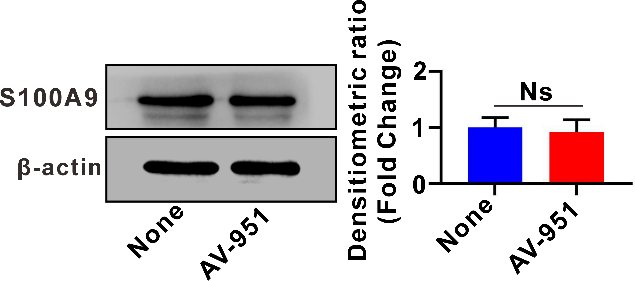


Fig. S6 Western blot analysis was performed to assess S100A9 expression in HuH-7 cells treated with or without AV-951 for 24 hours. Densitometric values of the protein bands were normalized to β-actin and compared to the control group. Quantitative results are presented in the right panel. Data are presented as mean ± SD. Ns, not significant. Statistical analysis of the data was performed using a Student’s t-test.
